# Effect of Environmental Temperatures on Proteome Composition of *Salmonella enterica* Serovar Typhimurium

**DOI:** 10.1016/j.mcpro.2022.100265

**Published:** 2022-07-02

**Authors:** Laura Elpers, Jörg Deiwick, Michael Hensel

**Affiliations:** 1 Abt. Mikrobiologie, Universität Osnabrück, Osnabrück, Germany; 2 CellNanOs – Center of Cellular Nanoanalytics Osnabrück, School of Biology/Chemistry, University of Osnabrück, Osnabrück, Germany

**Keywords:** cold adaptation, *Salmonella enterica*, bacterial pathogenesis, flagella, metabolism, H-NS, histone-like nucleoid structuring, IHF, integration host factor, LB, lysogeny broth, MS, mass spectrometry, NAPs, nucleoid-associated proteins, o/n, overnight, PCN, phosphate-carbon-nitrogen, SCV, *Salmonella*-containing vacuole, SPI, *Salmonella* pathogenicity island, STM, *Salmonella* enterica serovar Typhimurium, T1SS, type 1 secretion system, T3SS, type 3 secretion system

## Abstract

*Salmonella enterica* serovar Typhimurium (STM) is a major cause of gastroenteritis and transmitted by consumption of contaminated food. STM is associated to food originating from animals (pork, chicken, eggs) or plants (vegetables, fruits, nuts, and herbs). Infection of warm-blooded mammalian hosts by STM and the underlying complex regulatory network of virulence gene expression depend on various environmental conditions encountered in hosts. However, less is known about the proteome and possible regulatory networks for gene expression of STM outside the preferred host. Nutritional limitations and changes in temperature are the most obvious stresses outside the native host. Thus, we analyzed the proteome profile of STM grown in rich medium (LB medium) or minimal medium (PCN medium) at temperatures ranging from 8 °C to 37 °C. LB medium mimics the nutritional rich environment inside the host, whereas minimal PCN medium represents nutritional limitations outside the host, found during growth of fresh produce (field conditions). Further, the range of temperatures analyzed reflects conditions within natural hosts (37 °C), room temperature (20 °C), during growth under agricultural conditions (16 °C and 12 °C), and during food storage (8 °C). Implications of altered nutrient availability and growth temperature on STM proteomes were analyzed by HPLC/MS-MS and label-free quantification. Our study provides first insights into the complex adaptation of STM to various environmental temperatures, which allows STM not only to infect mammalian hosts but also to enter new infection routes that have been poorly studied so far. With the present dataset, global virulence factors, their impact on infection routes, and potential anti-infective strategies can now be investigated in detail. Especially, we were able to demonstrate functional flagella at 12 °C growth temperature for STM with an altered motility behavior.

## Highlights

• Systematic proteome analyses of STM grown at 37 °C compared to 20, 16, 12, 8 °C was performed.
• Altered gene ontology classes reveal global adaptation of environmental temperatures.
• Virulence proteins for interaction with mammalian hosts increased at 37 °C.
• STM build functional flagella during growth at 12 °C with altered motility.

## Graphical Abstract

**Figure:**
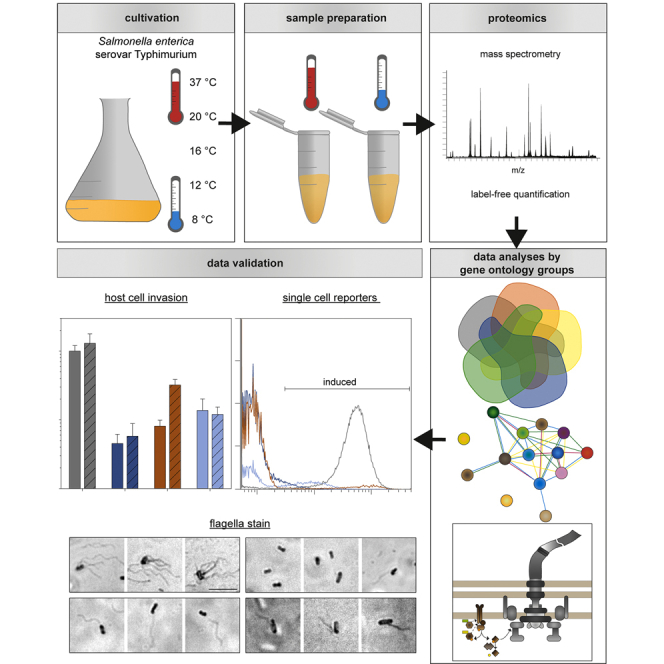

## In Brief

We performed systematic proteome analyses of *Salmonella enterica* serovar Typhimurium (STM) grown at human host body temperature (37 °C) compared to environmental temperatures relevant of agriculture (20, 16, 12, 8 °C). Altered abundance of members of various gene ontology classes reveal global adaptation of environmental temperatures. Virulence proteins for interaction with mammalian hosts were increased at 37 °C, while metabolic proteins were increased at lower temperatures. During growth at 12 °C, STM build functional flagella and showed altered motility.

*Salmonella enterica* serovar Typhimurium (STM) is a common causative agent of gastroenteritis worldwide. The foodborne pathogen is often associated with contaminated animal products such as pork, chicken, and eggs but also with contaminated nonanimal products such as vegetables, salads, fruits, herbs, and water (1, 2). Therefore, STM has to cope with changing conditions in various environments, leading to stress conditions originating from temperature, nutritional limitations, UV, oxygen radicals, osmolarity, or extreme pH. Changes in environmental conditions are encountered during cultivation of fresh produce (open field conditions), during colonization of abiotic surfaces (including equipment involved in food production but also hospital supplies), and during infection of various hosts. However, STM is able to survive a wide range of harsh environments, for example temperature changes. Some *Salmonella* strains are able to survive at least temperatures ranging from 2 °C up to 54 °C (3, 4). Complex regulatory networks allow the adaptation to changing environmental conditions, for example the cold shock response (reviewed in (5)).

Nevertheless, the optimal growth temperature is 37 °C, which is also present in the mammalian hosts. Many studies investigated virulence gene expression and gene regulation, especially during the process of infection, at a temperature of 37 °C (6, 7). For example, the coordinated process of gene expression to adapt to the acidic conditions in the stomach of mammals is shown (8). Further, the coordinated expression of virulence genes is induced by conditions present in the intestinal lumen, and coregulation of genes encoded in *Salmonella* pathogenicity island 1 (SPI1), SPI4, and the *flhDC* flagellar operon was observed (7, 9). SPI4 gene expression leads to synthesis and presence on the cell surface of the giant adhesin SiiE by its cognate type 1 secretion system (T1SS), mediating intimate contact to epithelial cells. Expression of SPI1 genes leads to the assembly of a type III secretion system (T3SS) and the translocation of effector proteins into the host cell (10). Effector proteins such as SipA, SopB, SopE cause the rearrangement of the host cell cytoskeleton resulting in membrane ruffle formation and the uptake of STM (11).

As many other gastrointestinal pathogens, STM deploys flagella-mediated motility that is coordinated by a complex sensory system involving various chemotaxis sensors. Flagella have multiple roles in host pathogen interactions (12), and for many bacterial pathogens, motile behavior is controlled by environmental temperature. While STM robustly expresses flagella at temperatures present in mammalian hosts, little is known about motility under environmental conditions.

Once inside the host cell, STM remains within a compartment termed *Salmonella*-containing vacuole (SCV), which is characterized by a low pH, nutritional limitations, and phosphate and magnesium (Mg^2+^) deprivation. These conditions present in the SCV induce the expression of SPI2 genes, encoding another T3SS and specific effector proteins, which are secreted directly into the host cell cytoplasm. Thereby, the host cell vesicle trafficking is manipulated and STM induces the formation of a network of *Salmonella*-induced filaments necessary for the survival and replication inside mammalian host cells (13, 14, 15). In addition, the metabolism of STM adapts to nutrient-restricted conditions inside the SCV (16). This complex gene regulation during pathogenesis enables STM to infect the mammalian host in a very efficient manner.

While these complex networks of gene regulation were investigated in great extent for STM grown at 37 °C (17), less is known about the expression of proteins and possible gene regulations at various environmental conditions. Here, nutritional limitations and temperature changes are the most obvious stresses outside the native host and during agricultural cultivation of various plants at field conditions. Therefore, we aim to unravel the proteome of STM grown in rich medium and minimal medium at various temperatures in comparison to the well-known proteome of STM grown at 37 °C by HPLC/MS-MS and label-free quantification. With this method, we are able to detect the impact of changes in medium composition and temperature on the complete proteome of STM. Thereby, changes in abundance of proteins associated with STM pathogenesis and potentially the presence of proteins under so far unknown environmental conditions are uncovered.

## Experimental Procedures

### Bacterial Strains and Culture Conditions

This study used STM NCTC 12023 as WT strain, and isogenic mutant strains as listed in supplemental Table S1 were used. Strains with deletions of *ssrAB*, *hilD*, or *flhDC* were created using λ Red recombination in STM WT harboring pWRG730. One-step gene inactivation was performed as described previously (18) using oligonucleotides listed in supplemental Table S2. Further deletion of *aph* from MvP2930 (Δ*hilD*::*aph*) was performed using pE-FLP encoding FLP recombinase as described (19). The deletions were confirmed by colony PCR using oligonucleotides listed in supplemental Table S2.

### Construction of Plasmids

Plasmids used in this study are listed in supplemental Table S3. Dual fluorescence reporter plasmid p4889 was used as basis vector and DNA fragments representing sequence 300 bp upstream of the translational start site harboring promoters of *ssaG*, *prgH*, *flhB*, or *motA* were amplified from STM genomic DNA using oligonucleotides listed in supplemental Table S2. DNA fragments were inserted in front of sfGFP in plasmid p4889 by Gibson Assembly. After confirming reporter functions, the LVA-tag was fused to the C-terminus of sfGFP to achieve increased degradation of GFP. LVA was added by site-directed mutagenesis (NEB) using the oligonucleotides p4889-LVA SDM For and sfGFP-LVA Rev (supplemental Table S2).

### Culture Conditions and Analysis of Growth Kinetics

Unless otherwise mentioned, bacteria were grown aerobic in LB (lysogeny broth) medium or PCN (phosphate-carbon-nitrogen) minimal medium or on agar plates containing the same media. LB is composed of 1% (w/v) bacto tryptone, 0.5% (w/v) bacto yeast extract, 0.5% (w/v) NaCl, adjusted to pH 7.4. PCN is MOPS-buffered salts minimal medium pH 7.4, 1 mM phosphate, 0.4% (w/v) glucose (20). Media components and further reagents are listed in supplemental Table S4.

For analyses of growth kinetics, overnight (o/n) cultures of STM WT grown in LB or PCN media at 37 °C were used. A_595_ were determined and subcultures of 100 ml fresh LB medium or PCN medium in 500 ml baffled flasks inoculated to A_595_ = 0.01. Incubation occurred in an incubator cabinet (Innova42, Eppendorf) at temperatures ranging from 8 °C to 37 °C with orbital agitation at 160 rpm. A_595_ was measured hourly for 24 h, followed by longer intervals.

### Analyses of Motility on Swim Agar Plates

Swim agar plates contained 0.3% (w/v) bacto agar and either LB or PCN medium, that is, MOPS-buffered salts minimal medium, pH 7.4, 1 mM phosphate, 0.4% (w/v) glucose (20). The centers of plates were inoculated with 1 μl subculture and cultures were grown under the same conditions used for cultures grown for proteomic analyses. Swim agar plates were incubated at 12 °C or 37 °C right-side-up in a humid chamber to prevent desiccation. Swim ring diameters were determined and images were taken at various time points.

### Infection of Polarized Epithelial Cells

The canine polarized epithelial cell line MDCK was used for analyses of host cell invasion by STM. STM WT was cultured in LB or PCN media at 37 °C or 12 °C as for proteome analyses. After addition of bacterial inoculum to host cell cultures, the assays were directly incubated for 30 min to allow adhesion and invasion (static), or assays were subjected to centrifugation for 5 min at 500*g* prior incubation to increase contact of STM to host cells and to compensate for reduced motility. Noninternalized STM were killed by addition of Gentamicin, intracellular STM were quantified by determination of colony-forming units on agar plates after lysis of host cells as described before (7).

### Protein Extraction of Whole Cells

Bacteria at late–log phase were investigated by proteome analyses. For analyses of the entire proteome, lysates of whole cells were prepared. Therefore, cells of 3 ml bacterial culture were pelleted and resuspended in 200 μl urea (9.3 M in 50 mM Tris–HCl buffer, pH 8.0). The bacterial lysate was incubated for 1 h at 37 °C with shaking and stored at −80 °C for at least 16 h. The lysates were thawed and cell debris were removed by centrifugation at 20,000*g* for 10 min using an Optima MAX Ultracentrifuge (Beckman Coulter) and rotor TLA 100.3. Supernatant was used for protein digestion and proteomic analyses.

### Protein Digestion

Protein concentration was determined using a NanoPhotometer (IMPLEN). For each sample, 100 μl were digested. Reduction occurred by 5 mM DTT in ABC buffer (50 mM ammonium bicarbonate/NH_3_, pH 8.5) for 30 min at 37 °C. For alkylation, 15 mM iodoacetamide in ABC buffer were added to samples and incubated for 30 min at RT in the dark. For digestion, 10 μg protein was digested by 0.3 μg LysC/Trypsin (Promega) for 3 h at 37 °C. ABC buffer was added to a final volume of 79.2 μl and further incubated o/n at 37 °C. The reaction was stopped by adding 100% (v/v) formic acid to a final concentration of 1% (v/v) formic acid. Remaining particles were removed by centrifugation and the peptide-containing supernatant was transferred to HPLC vials and subjected to mass spectrometry (MS) measuring 1 μg digested protein.

### Label-Free Protein Quantification by MS

HPLC/MS-MS analyses were done using an Ultimate 3000 Nano HPLC (ThermoFisher). Therefore, 8 μl of samples were desalted and concentrated using a precolumn (ThermoFisher, C18 PepMap 5 μm, 100 A with dimension of 300 μm (id) x 5 mm (length)). The corresponding solvent was water supplemented with 0.1% TFA/H_2_O at a flow rate of 25 μl/min. The loaded precolumn was switched into the `nano flow line´ (250 nl/min) with an Easy Spray column (ThermoFisher, PepMap RSLC C18, 2 μm, 100 A with dimension of 75 μm (id) × 500 mm). Peptides were eluted by 80% acetonitrile and 0.1% in H_2_O continuously in 180 min. Electrospray ionization was done at 1500 V (ESI Spray Source, ThermoFisher). A Q-Exactive Plus orbitrap mass spectrometer (ThermoFisher) was used to determine the MS/MS data under conditions described in supplemental Table S5.

### Data Analysis

The resulting spectra were analyzed by Peaks Studio X (version 106, Bioinformatics Solution Inc) with data refinement including precursor correction and chimera scan followed by *de novo* analysis (21). The peptides were identified using a *de novo* approach and the corresponding proteins by a DB search using a database for STM (strain ATCC 14028s/SGSC 2262; 5369 proteins; Uniprot, 21.07.2019, (22)). Peptides were digested with LysC and trypsin generating lysine and arginine containing peptides and two missed cleavage was conceded. For DB searches, the strict mode was applied excluding any terminus to disobey enzyme specificity. For both searches, the MS tolerance was adjusted to 10.0 ppm, the MS/MS tolerance to 0.2 Da posttranslational variable modifications carbamidomethylation of cysteines and oxidation of methionine were chosen. In addition, label-free quantification was performed using three biological replicates with a retention time shift of 25.0 min and a false discovery rate of 1% based on the decoy fusion approach (22). Proteomic data were subsequently analyzed for potential altered gene groups by comparison to gene ontology groups. The mass spectrometry proteomics data have been deposited to the ProteomeXchange Consortium via the PRIDE (23) partner repository with the DB included as STM14028_5.fasta. For visualization by Venn diagrams, the tool http://bioinformatics.psb.ugent.be/webtools/Venn/ was used.

### Experimental Design and Statistical Rationale

Ten different samples (2 media with five different growth temperatures) were analyzed with three biological replicates each (n = 3). Protein abundances were compared to 37 °C cultures in the respective medium as control. All three biological replicates were subjected to comparative analyses in Peaks Studio X, and outliers were repeated ensuring analyses with comparable score distributions. Further data were normalized to house-keeping protein DnaK of reference sample STM grown in LB medium or PCN medium at 37 °C. For determination of significantly differential protein abundances in STM grown at various temperatures compared to STM grown at 37 °C in the respective medium, we used Student´s *t* test, considering *p* < 0.05 as significant, and the Benjamini-Hochberg correction to adjust for multiple hypothesis testing (24). Further, only proteins with at least > 2-fold change in abundance and presence in all three replicates in both groups with at least one identical peptide found were included. In addition, proteins were analyzed with regard to their exclusive occurrence in only one of two compared groups. Hence, the protein has to be found in all three replicates of the respective group.

### Visualization of Flagella

Bacterial subcultures were diluted 1:2 in PBS and 10 μl of suspensions were applied on a glass slide and covered by a 24 mm coverslip. After allowing to dry for 10 min, 5 μl of flagella stain were carefully applied to the border of the coverslip to ensure absorption of the flagella stain. Flagella stain was modified Ryu mordant (25) consisting of solution 1: 20% tannic acid in saturated aqueous solution of AlK(SO_4_)_2_ ∗ 12 H_2_O, and solution 2: 12% (w/v) crystal violet in 95% EtOH; 10 parts of solution 1 and one part of solution 2 were mixed, filtered through 0.2 μm pore filter, and stored in small aliquots at RT. Flagella stain was added to bacterial suspensions under a coverslip and dried for approx. 15 min. Stained bacteria were imaged directly using a Zeiss Axio Lab A1 and Bresser MicroCam SP 3.1 using a 100× oil objective. Images were further processed with MicroCamLab II, version x64, 3.7.8752.

### Reporter Analyses by Flow Cytometry

Bacteria were grown in LB medium, PCN medium (1 mM P_i_, pH 7.4), or PCN medium (0.4 mM P_i_, pH 5.8) o/n. Incubation occurred in 20 ml fresh LB medium or PCN medium in 100 ml baffled flasks inoculated to A_595_ = 0.01. Cultures were grown in an incubator cabinet (Innova42, Eppendorf) at 37 °C or 12 °C with orbital agitation at 160 rpm according to incubation times used for proteomic analyses. Bacteria were fixed in 3.7% (w/v) formaldehyde in H_2_O_dd_ for 30 min at RT and further diluted in PBS for flow cytometry analyses using the Attune N × T Cytometer (ThermoFisher) with AttuneTM N × T Software (v4.2.0) basically as described before (26). For each analysis, 50,000 bacteria were measured. The strain deficient in respective regulation gene with plasmid was used as negative control for gating and set to <1% positive cells (supplemental Fig. S6). Flow cytometry analyses were done for three biological replicates with four technical measurements.

## Results and Discussion

We investigated the proteomes of STM grown at various temperatures and used two distinct media mimicking a nutritional rich environment (LB medium) and a minimal medium supplemented with glucose (PCN medium) mimicking a nutritional limited environment. Further, STM was grown at 37 °C as temperature of mammalian hosts (27), 20 °C as room temperature and growth temperature of various vegetables and salad species (28, 29), 16 °C as growth temperature of various vegetables and salad species (28, 29), 12 °C as upper limit of average temperature in Europe, and often minimal temperature for growth of salad species (28, 29), and 8 °C as lowest temperature for STM growth, potential storage temperature of food, and lower limit of average temperature in Europe (28, 30).

### Growth Kinetics of *S. Typhimurium* in LB and PCN Media at Various Temperatures

Prior to proteomic analyses, the growth kinetics of STM were determined under the specified conditions for media and temperature. An o/n culture grown in respective medium at 37 °C was used to inoculate subcultures in same media for growth at various temperatures. Growth was determined by A_595_ measurements until no further increase was observed (supplemental Fig. S1). Since previous studies showed the highest level of invasion of nonphagocytic mammalian cells for STM subcultured to late–log phase (31, 32, 33), we decided to use cultures of this growth phase for all media and temperature conditions to harvest bacteria for proteomics analyses. The standard laboratory growth conditions (LB broth, 37 °C) for STM reached late-log growth phase in 100 ml medium in 500 ml flasks with two baffles at 160 rpm after 5 h, whereas STM growth in PCN medium was slower and late–log phase was reached after 9 h of growth. Growth kinetics were only determined once. However, decrease of temperature led to higher generation times and increase of cultivation time to reach late–log phase (supplemental Fig. S1 and supplemental Table S6). Hence, STM was grown in LB medium at 20 °C, 16 °C, 12 °C, and 8 °C in LB medium for 20 h, 24 h, 56 h, and 168 h, respectively (supplemental Fig. S1, squares). STM was grown in PCN medium at 20 °C, 16 °C, 12 °C, and 8 °C in PCN medium for 23 h, 32 h, 61 h, and 244 h, respectively (supplemental Fig. S1, circles).

### Proteome Composition of STM Whole Cells Grown in Rich and Minimal Medium at Various Temperatures

Via label-free quantification, relative differences in protein abundances in STM grown at various temperatures compared to STM grown at 37 °C in the same medium were calculated (supplemental Tables S7 and S8). For statistical analyses, only proteins were included which were detected in all three biological replicates in both samples with at least one identical peptide and considering *p* < 0.05 as significant. Further, proteins were analyzed with regard to their exclusive occurrence in only one of two compared samples. These unique proteins had to be found in all three replicates in the respective group. Identified proteins and comparisons of relative differences in protein abundances at various temperatures compared to STM grown at 37 °C in the same medium are available at ProteomeXchange Consortium via the PRIDE partner repository (see ‘Data Availability’). Subsequently, sorting proteins with differential abundances according to gene ontologies revealed potential altered functional classes and metabolic pathways which were further analyzed (34, 35). For detailed information on label-free quantification and proteomic data analyses, see ‘Label-Free Protein Quantification by Mass Spectrometry’ and ‘Experimental Design and Statistical Rationale’ in section ‘Experimental Procedures’. In total, 978 to 1463 proteins were found in STM grown in LB media at various temperatures, whereas protein numbers of STM grown in PCN media were lower (616–1336 proteins) (supplemental Table S7). Typical proteins, that is, temperature-related proteins (heat and cold shock proteins) were found for STM grown at the respective temperature representing the reliability of the analyzed samples.

### Abundance of Nucleoid-Associated Proteins is Dependent on Medium and Growth Temperature

**Fig. 1:**
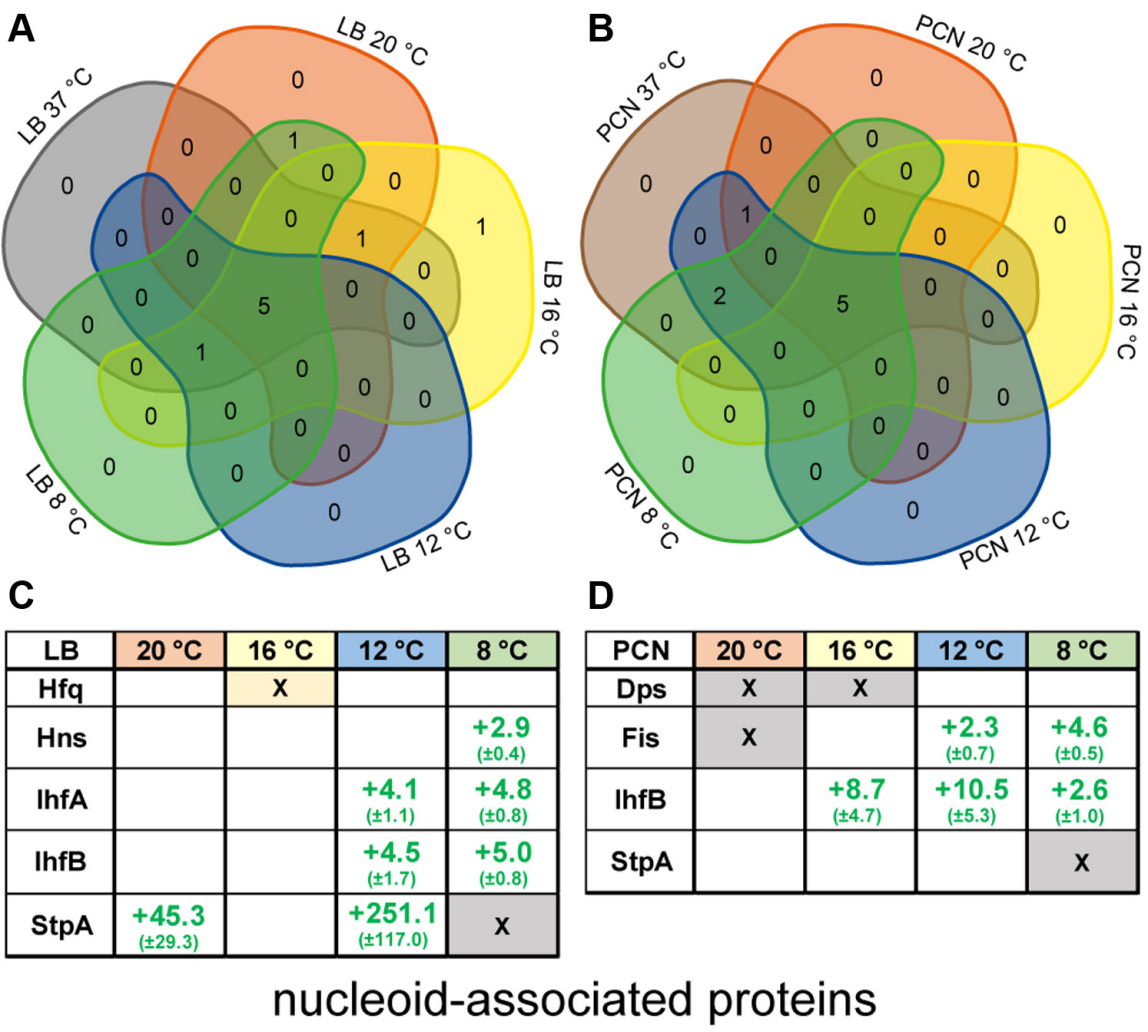
**Differentially abundant nucleoid-associated proteins in STM grown in LB or PCN medium at various temperatures.** Proteins were analyzed with regard to their occurrence in the compared groups. Proteomic data were subsequently analyzed by comparison to gene ontology groups (34, 35), as shown here for number of nucleoid-associated proteins (NAPs) in Venn diagrams (*A*, LB; *B*, PCN; supplemental Table S10). Differential protein abundances in STM grown at various temperatures compared to STM grown at 37 °C in the respective media were statistically analyzed using the Student´s *t* test (*p* < 0.05) and the Benjamini-Hochberg correction. Only proteins were included which showed at least 2-fold change in abundance. Shown are abundances of NAPs in STM cultured in LB (*C*) or PCN medium (*D*) with detailed changes of protein levels depicted by *green* font for higher abundance in STM grown at 37 °C (figures indicate x-fold change of protein abundance). SDs for proteins abundances of the three replicates are given in brackets. X-marked cells indicate the presence of protein unique in STM grown at 37 °C (*gray*) or various lower temperatures (colors). LB, lysogeny broth; STM, *Salmonella enterica* serovar Typhimurium.

In addition to several proteins involved in regulation of gene expression, bacteria encode for nucleoid-associated proteins (NAPs) which bind to the bacterial nucleoid thereby contributing to chromosome packaging within the cell. Further NAPs are also known for binding RNA influencing the gene expression profile. For *Salmonella* and *Escherichia coli*, a subset of major NAPs is known: IHF, H-NS, Fis, Hfq, StpA, and Dps (36, 37). Studies revealed the importance of NAPs in gene expression of several virulence factors and in global alteration of the proteome (38). Here, we observed changes in the overall presence and in the abundance of NAPs in STM dependent on growth temperature and medium (Fig. 1). The integration host factor (IHF) consisting of IhfA and IhfB forming a dimer was found to be significantly higher in STM grown at 37 °C in LB medium than grown at 12 ° and 8 °C (Fig. 1*C*). Comparison of STM grown at 16 °C revealed higher but nonsignificant abundances for IhfA (2.8-fold) and IhfB (3.2-fold), whereas no changes were observed for STM grown at 20 °C compared to 37 °C. Mangan *et al*. (39) showed by real time-PCR decreased expression of SPI1, SPI2, SPI4, and flagellar genes in a *Salmonella* IHF mutant strain (Δ*ihfA* Δ*ihfB*), thereby indicating the requirement of IHF in virulence gene expression. These observations were further supported by decreased ability of the IHF mutant strain to invade Chinese hamster ovarian cells and Caco-2 cells (human colon carcinoma cells) compared to *Salmonella* WT. IHF was further shown to be involved in the induction of SPI1 gene expression. H-NS (histone-like nucleoid structuring, encoded by *hns*) represses expression of SPI1 genes due to repression of *hilA* expression, potentially by binding DNA and promoting looping which was shown by atomic force microscopy (40). However, IHF binds a regulatory region in front of *hilA*, thus preventing H-NS binding, which results in the expression of HilA and subsequently SPI1 genes (41). Moreover, deletion of *ihfA ihfB* resulted in decreased biofilm formation, less Curli fimbriae and cellulose production in *Salmonella Enteritis* (42) indicating a global role in virulence gene expression. Proteomic analyses done in this study further suggest the role of IhfA and IhfB in the virulence protein profile since higher abundance of virulence-associated proteins (including SPI1-encoded, SPI4-encoded, and flagellar proteins) was observed for STM grown in LB at 37 °C which is in line with higher abundances of IhfA and IhfB at 37 °C. Nevertheless, STM grown in PCN medium at 37 °C exhibited higher abundance of IhfB than STM grown at temperatures ranging from 8 °C to 16 °C in PCN, whereas no difference was observed for 20 °C (Fig. 1*D*). In addition, IhfA was found in similar abundance in STM at all growth temperatures. Since IHF is described as heterodimer of IhfA and IhfB (37), no conclusion on functionality can be made if only one IHF protein is identified in higher abundance.

The paralog of H-NS, StpA (suppressor of T4 td mutant phenotype A), was found in higher abundance in STM grown in LB medium at 37 °C in comparison to all other investigated temperatures, though changes were not significant for STM grown at 16 °C (Fig. 1*C*). Comparison of STM grown at 37 °C to 12 °C revealed a 251.1-fold (±117.0) increased abundance of StpA, whereas StpA is completely absent in STM grown at 8 °C. StpA exhibits similar functions as H-NS as a result of direct protein–protein interactions of both proteins (shown for *E*. *coli* (43)) thus leading to a potential involvement of StpA in H-NS–mediated DNA binding for global gene regulation. Nonetheless, proteomic data showed no altered abundance of H-NS due to changes of growth temperatures.

NAPs seem to have important functions in virulence protein profile but are additionally involved in regulation of all other bacterial genes, since they affect chromosome packaging in a global manner. Therefore, NAPs are of special interest in global protein changes but their specific impact on various regulation pathways are difficult to elucidate.

### Abundance of Stress Response Proteins is Dependent on Growth Temperature

**Fig. 2:**
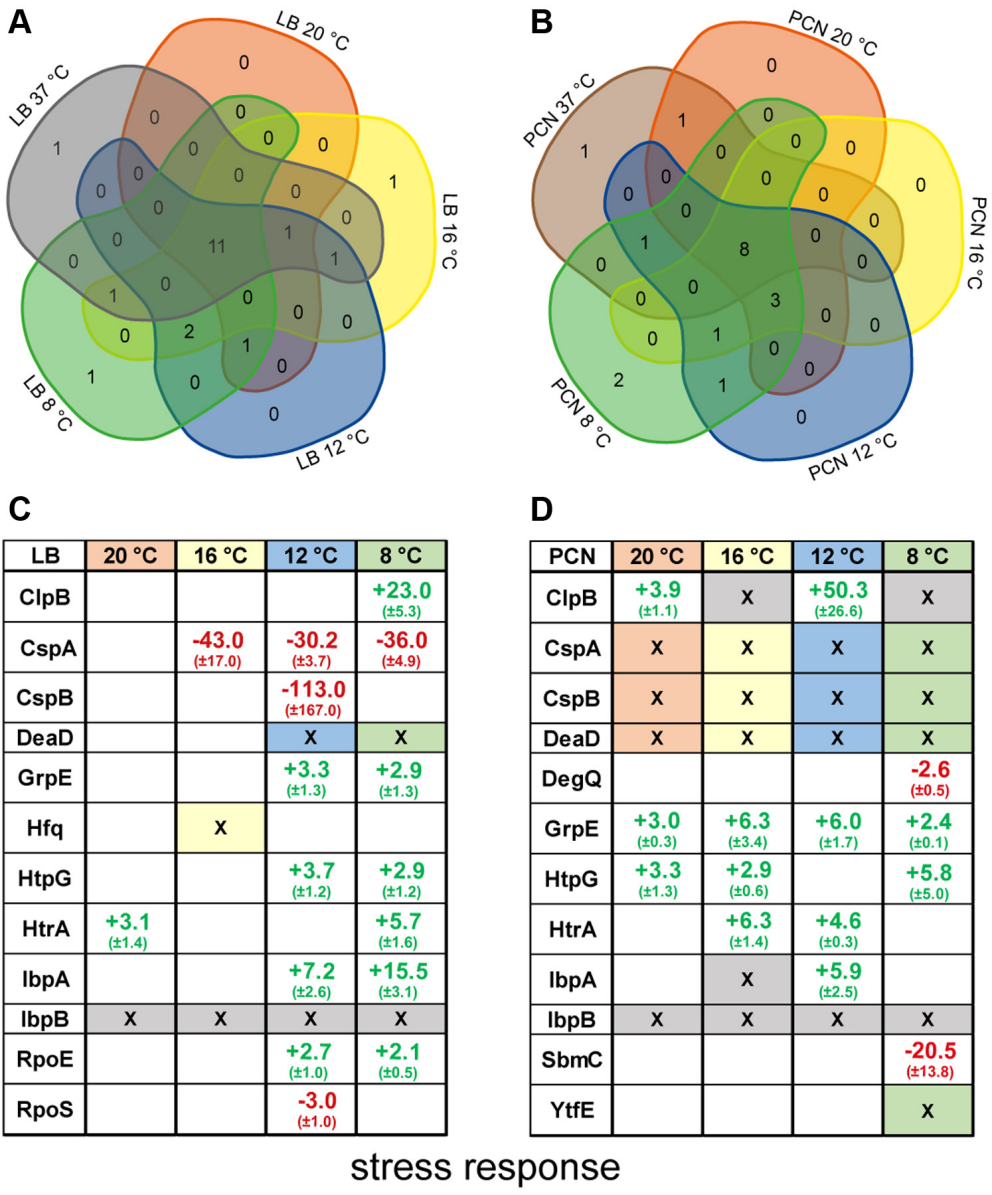
**Differentially abundant proteins involved in stress response in STM grown in LB or PCN medium at various temperatures.** Proteins were analyzed regarding their occurrence in the compared groups. Proteomic data were subsequently analyzed by comparison to gene ontology groups (34, 35), as shown here for numbers of stress response–associated proteins in Venn diagrams (*A*, LB; *B*, PCN; supplemental Table S10). Details of changes of protein levels are shown in (*C*) and (*D*). *Green font* indicates higher abundance in STM grown in LB medium at 37 °C; *red font* indicates *lower abundance* in STM grown at 37 °C compared to various lower growth temperatures or to STM grown in PCN medium at 37 °C (figures indicate x-fold change of protein abundance). X-marked cells indicate unique presence of a protein in STM grown at 37 °C (*gray*), or the respective lower temperature, or PCN medium at 37 °C (colors). For information on legend and statistical analyses performed, see Figure 1. LB, lysogeny broth; STM, *Salmonella enterica* serovar Typhimurium.

In response to nutritional limitations and growth temperature, often stress proteins are upregulated to protect the bacterial cell. STM grown in LB medium at different growth temperatures revealed the increased abundance of several proteins associated with stress responses particularly at 37 °C in comparison to lower growth temperatures. The strongest increase in abundance of six stress response proteins and synthesis of one unique protein was observed for STM grown in LB medium at 37 °C compared to 8 °C (Fig. 2, *A* and *C*). These proteins include, among others (GrpE, HtpG, HtrA, RpoE), ClpB (23.0-fold ±5.34; chaperone protein) and IbpA (15.5-fold ±3.1; small heat shock protein), as well as IbpB (small heat shock protein) being the one unique protein present in 37 °C compared to all other temperatures.

The synthesis of stress response proteins in STM grown at lower temperatures appears to be more selective. Amount of CspA (cold shock protein A) is highly increased from 8 °C to 16 °C, and DeaD (ATP-dependent RNA helicase; also called CsdA) was only detected at temperatures below 12 °C. In STM grown in LB medium at 20 °C, no altered protein abundance of stress response proteins was observed indicating a comparable stress level to STM grown in LB medium at 37 °C. However, stress response proteins are already present in STM grown in PCN medium at higher growth temperatures (Fig. 2, *B* and *D*). Thus, CspA, CspB (cold shock protein B), and DeaD are found at temperatures ≤20 °C but not in STM grown at 37 °C in PCN medium, indicating an important feature of those proteins at lower temperatures under nutritional limitations. In comparison, IbpB is found solely in STM grown at 37 °C, as well as ClpB in comparison to 16 °C and 8 °C, further abundance of ClpB is increased in 37 °C compared to 20 °C (3.9-fold ±1.1) and 12 °C (50.3-fold ±26.6). The amount of GrpE (heat shock protein-70 cofactor) is increased in STM grown at 37 °C compared to all other temperatures, as well as HtpG (chaperone protein) in comparison to STM grown at 20 °C, 16 °C, and 8 °C. Stress response proteins with different abundances or unique presence in STM grown at 37 °C in LB or PCN medium (GrpE, ClpB, IbpA, IbpB, and HtpG) are all proteins with chaperone function. Thereby GrpE is part of the DnaK–DnaJ chaperone complex for proper protein folding. Less is known about specific conditions of GrpE synthesis, but it was shown that DnaK/DnaJ levels are temperature-dependent controlled by an RNA thermometer in front of *dnaJ*, leading to higher amounts of DnaJ at higher temperatures (37 °C, (44)). Further, HtpG, a homolog to eukaryotic heat shock protein 90, is involved in the stabilization and maturation of proteins (45). ClpB is another chaperone responsible for protein disaggregation and refolding and is necessary for survival under several stresses, that is, heat stress at 42 °C, oxidative stress induced by paraquat (1,1-dimethyl-4,4-bipyridinium) and hypochlorous acid (HClO) (46). IbpA and IbpB are encoded by genes in an operon and belong to the family of small heat shock proteins (sHsp). The 5′-UTR of *ibpA* encodes a *repression of heat shock gene expression*-like RNA thermometer allowing temperature-controlled expression. IbpAB are responsible for the binding of aggregated proteins, facilitating survival at higher temperatures (47). Therefore, all stress-related proteins detected in higher abundance in STM at increasing temperature are chaperones related to heat stress. Thus, the optimal growth temperature of 37 °C leads to the highest replication rate and therefore to intensive protein biosynthesis, requiring high amounts of chaperones to guarantee correct protein folding.

Proteins involved in stress response to lower temperatures and cold shock were specifically found in STM grown at temperatures from 8 to 20 °C in LB and PCN medium. We mainly detected CspA, CspB, and DeaD being present in higher amounts in STM at lower temperatures. CspA is the best-studied cold shock protein in *E. coli* and *Salmonella*, and several studies showed that the *cspA* mRNA adapts to changes in temperature (potentially related to hairpin formation), which finally lead to more efficient translation of *cspA* mRNA at lower temperatures. CspA functions as RNA chaperone, preventing RNA from misfolding (48). Similar to CspA, CspB is present at lower temperatures and may have a function similar as CspA (49). The protein DeaD acts as RNA helicase involved in various cellular processes at lower temperatures including ribosome biogenesis, mRNA degradation, and the initiation of translation (50). Therefore, STM adapts to lower temperatures by specified chaperones necessary for the proper folding of proteins and protection of mRNA from misfolding during cold stress.

### Changes in Metabolic Pathways are Dependent on Medium and Growth Temperature

**Fig. 3:**
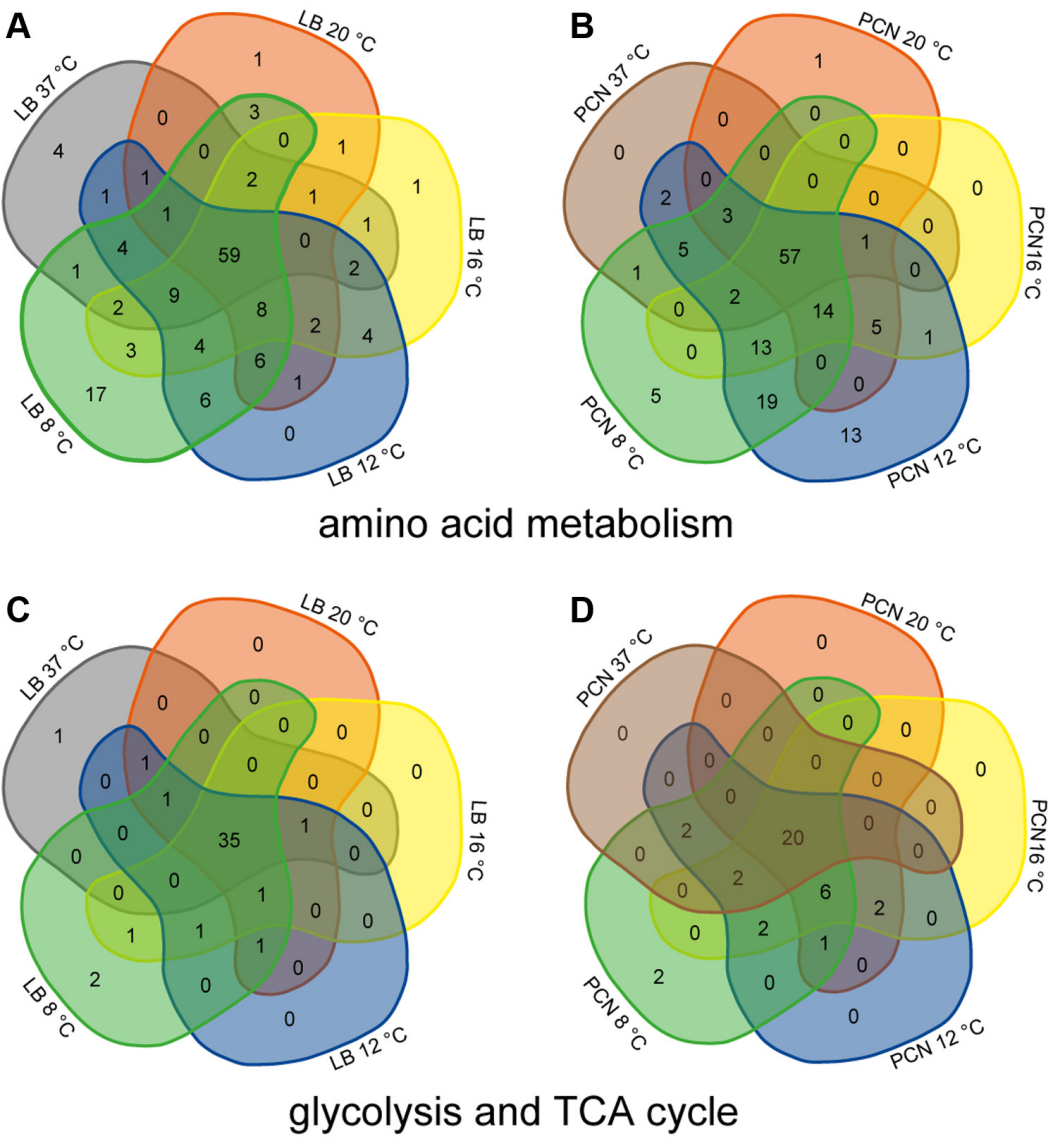
**Differentially abundant enzymes of amino acid metabolism, glycolysis, or TCA cycle in STM grown in LB or PCN medium at various temperatures.** Proteins were analyzed regarding their occurrence in the compared groups. Proteomic data were subsequently analyzed by comparison to gene ontology groups (34, 35), as shown here for numbers of proteins associated with the amino acid metabolism (*A*, LB; *B*, PCN; supplemental Table S10) and glycolysis and TCA cycle (*C*, LB; *D*, PCN; supplemental Table S10) in Venn diagrams. Color coding and statistical analyses as described for Figure 1. LB, lysogeny broth; STM, *Salmonella enterica* serovar Typhimurium.

Changes in growth temperature, independent of the selected medium used, resulted in a remarkable altered protein composition involved in metabolic pathways. Sorting of proteins according to gene ontologies revealed changes for many proteins involved in amino acid metabolism in STM grown under decreased temperature conditions compared to STM grown at 37 °C (Fig. 3, *A* and *B*). STM grown in LB at 8 °C showed highest unique presence of 17 proteins involved in amino acid metabolism in comparison to all other temperatures. However, changes in protein abundances cannot be traced back to specific amino acid pathways, indicating a rather global change in amino acid metabolism. Increased abundances of proteins of amino acid metabolism of STM were also observed by Shah *et al*. (51) after short cold shock (30 min at 5 °C) and after exposition to continuous cold stress (5 h to 336 h at 5 °C). In contrast, STM grown in PCN at 12 °C showed unique presence of 13 proteins and an overall increased number of proteins associated to amino acid metabolism compared to all other growth temperatures.

Changes in the amino acid metabolism of STM grown in PCN medium are reasoned by the requirement to synthesize all amino acids from the sole C-source glucose, whereas in LB medium, amino acids are present in addition to carbohydrates. Therefore, distinct enzymatic equipment was anticipated. However, there was no correlation of increased or decreased protein abundances to specific biological processes or functions. These facts are reflected by the comparison of STM grown at 37 °C in LB medium to PCN medium (supplemental Fig. S2). Many proteins involved in amino acid metabolism are either found only in STM grown in LB medium (27 proteins) or PCN medium (15 proteins). Furthermore, some proteins are abundant in LB medium (15 proteins), and some proteins are present in lower amounts in LB medium (6 proteins) than PCN medium. These findings are in line with previous studies revealing altered amino acid biosynthesis in STM SL1344 (52) and in *E. coli* (53) in LB medium compared to minimal medium. Therefore, differential regulation of proteins involved in amino acid metabolism represents a common mechanism of bacteria to react to nutritional limitations.

In addition, an increase in numbers of differential protein abundances with decrease of temperature in STM grown in LB or PCN medium was also observed for proteins involved in the glycolysis and TCA cycle (Fig. 3, *C* and *D*). This gives further hints to metabolic changes upon temperature changes. For STM grown in LB medium, a core set of 35 proteins were found for all growth temperatures and only two proteins were uniquely found for STM grown in LB at 8 °C (Fig. 3*C*). This observation was also made for STM grown in PCN medium with a core set of 20 proteins for all growth temperatures (Fig. 3*D*).

In addition to changes in amino acid metabolism, Shah *et al*. (51) also observed an increased abundance of proteins involved in the TCA cycle in STM after cold shock (30 min at 5 °C). A previous study by real-time PCR on *E. coli* K-12 grown in minimal medium at various temperatures reported higher expression of glycolysis genes at lower temperatures than 37 °C (54). Another study revealed a link between growth temperature of 8 °C and higher abundance of TCA cycle enzymes in STM grown in tryptic soy broth medium at mid–log phase (55). As discussed in the mentioned studies, we hypothesize that the altered protein composition and increased number of proteins at lower temperatures in both media are necessary to compensate temperature-dependent decreased efficiency of glycolysis, TCA cycle, and amino acid metabolism, and to maintain enough energy for survival.

**Fig. 4:**
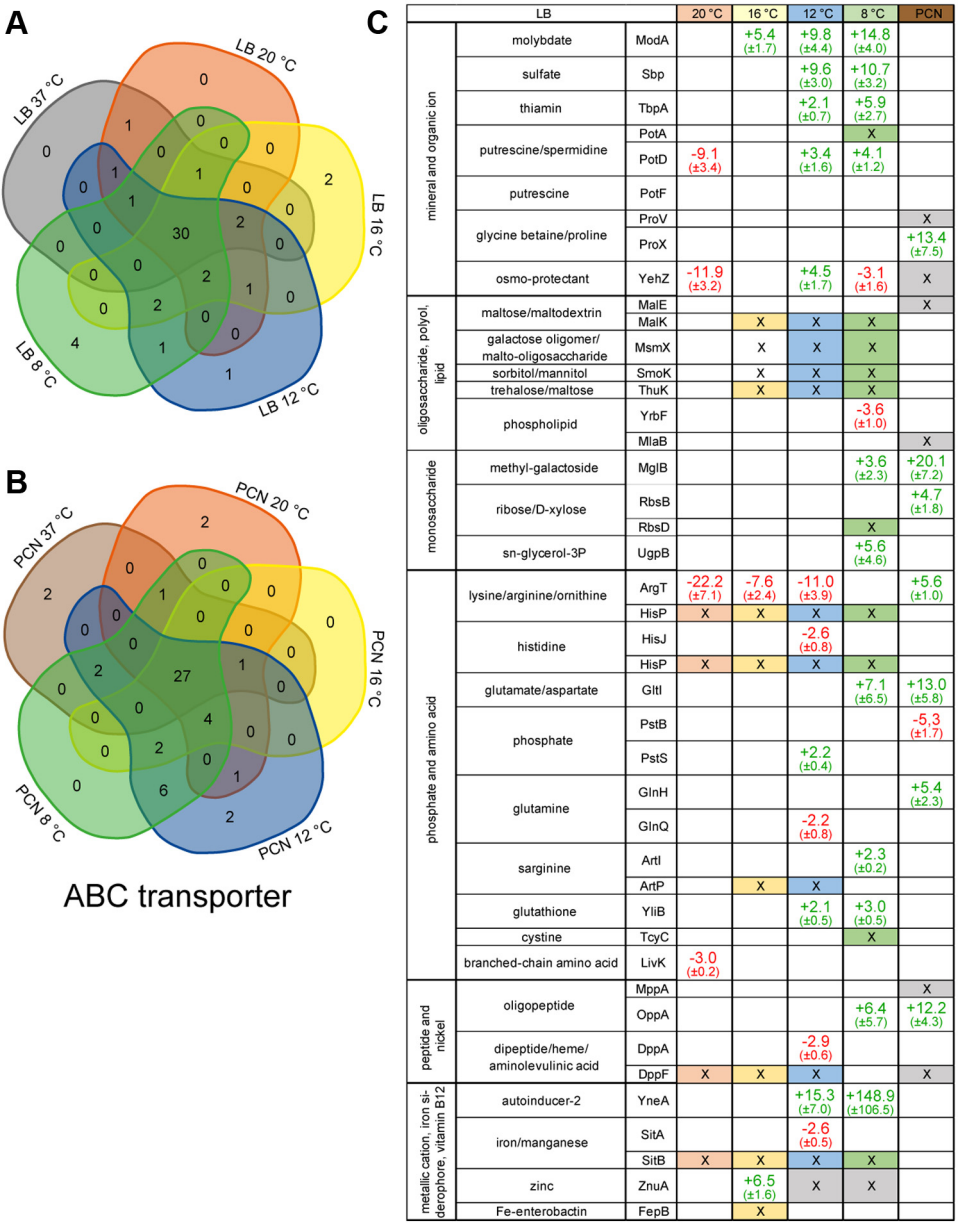
**Differentially abundant ABC transporters in STM grown in LB or PCN medium at various temperatures.** Proteins were analyzed regarding their occurrence in the compared groups. Proteomic data were subsequently analyzed by comparison to gene ontology groups (34, 35) and as shown for ABC transporter grouped according to KEGG Mapper (79) in Venn diagrams (*A*, LB; *B*, PCN; supplemental Table S10). *C*, the x-fold changes of protein levels of ABC transporters and associated proteins were determined. Figures indicate x-fold change of protein abundance ±SD. *Green or red font* indicate higher or lower abundance, respectively, in STM grown in LB medium at 37 °C compared to LB culture at 20 °C, 16 °C, 12 °C, or 8 °C or PCN medium at 37 °C. Cells marked by X indicate the presence of the protein uniquely in STM grown at 37 °C (*gray*) or at 20 °C, 16 °C, 12 °C, or 8 °C, or PCN medium at 37 °C (colors). See Figure 1 for information on legend and statistical analyses performed. LB, lysogeny broth; STM, *Salmonella enterica* serovar Typhimurium.

In addition to altered amino acid metabolism, glycolysis, and TCA cycle, changes in abundance of ABC transporters were observed (Fig. 4). Comparison of STM grown in LB medium at 37 °C to PCN medium at 37 °C showed higher abundances of ABC transporters or the unique presence of proteins of ABC transporters in STM in LB medium. However, in most cases due to the method chosen, information on membrane proteins of ABC transporters are often lacking. The ABC transporter protein with highest difference in abundance than STM grown in LB medium at 8 °C was YneA (also called LsrB), as part of the autoinducer 2 (AI-2) system (148.9-fold ±106.51, increased protein abundance in STM grown at 37 °C). This ABC transporter is formed by LsrACD and YneA, thereby, YneA binds AI-2 directly leading to Quorum sensing as a form of cell-to-cell communication of bacteria to regulate density-dependent gene expression (56, 57). Increased signaling activity was observed for *Salmonella* in presence of glucose, after growth in medium with high osmolarity (0.4 M NaCl) or at low pH (pH 5.0) representing the transition to host infection (58). Therefore, protein YneA might also be involved at higher temperatures representing temperature of various hosts (37 °C) as observed here.

ABC transporters for the uptake of oligopeptides (MppA) and glycine betaine/proline (ProV) were solely present in 37 °C cultures, further ABC transporters of oligopeptides uptake (OppA), glutamate/aspartate (GltI), methylgalactoside (MglB), and glycine betaine/proline (ProX) were increased in 37 °C cultures by factors of 10.3, 9.33, 18.24, and 7.23, respectively. Since LB medium contains various nutrient sources, *Salmonella* can easily procure their acquisition by ABC transporters, rather than synthesis with higher energy demand. Higher abundances of proteins involved in ABC transporters are commonly regulated by sensing the respective nutrient.

In contrast, STM in PCN medium has to synthesize metabolic intermediates *de novo*, therefore, ABC transporters are not required (52). In line with phosphate starvation in PCN medium, subunit PstB of the ABC transporter responsible for high affinity phosphate uptake is increased (5.3-fold ±1.74) in STM grown in PCN medium. In comparison to STM grown in LB medium at various temperatures, protein abundances of ABC transporter were different compared to STM grown at 37 °C. Abundances of ABC transporter subunits for uptake of sulfate, molybdate, thiamine, or putrescine/spermidine in STM grown in LB at 37 °C compared to 12 °C and 8 °C. Moreover, subunits of ABC transporters for iron/manganese, dipeptides, histidine, lysine/arginine/ornithine, and maltose/galactose/sorbitol/trehalose are often unique present in STM in LB medium grown at lower temperatures (20 °C to 8 °C). Higher abundance of amino acid transporter, that is, for lysine and arginine was observed before for cold-stressed *Salmonella* (51).

In both media, decreased temperatures were related to the further increase of relative abundant proteins involved in amino acids metabolism compared to STM grown at 37 °C. Possible reasons would be effects of temperature on efficiency of amino acid metabolism due to distinct temperature optima and compensation by higher levels of certain proteins involved.

### Abundance of Flagellar and Chemotaxis Proteins is Dependent on Medium and Growth Temperature

In addition to temperature-dependent changes in metabolism and stress response, the proteomic analyses in this study indicate the temperature-dependent synthesis of flagellar and chemotaxis proteins enabling a directed motility and is of crucial importance for *Salmonella* virulence. Several studies revealed the involvement of flagella-mediated motility in *Salmonella* infection of mammalian and plant hosts (59, 60).

**Fig. 5:**
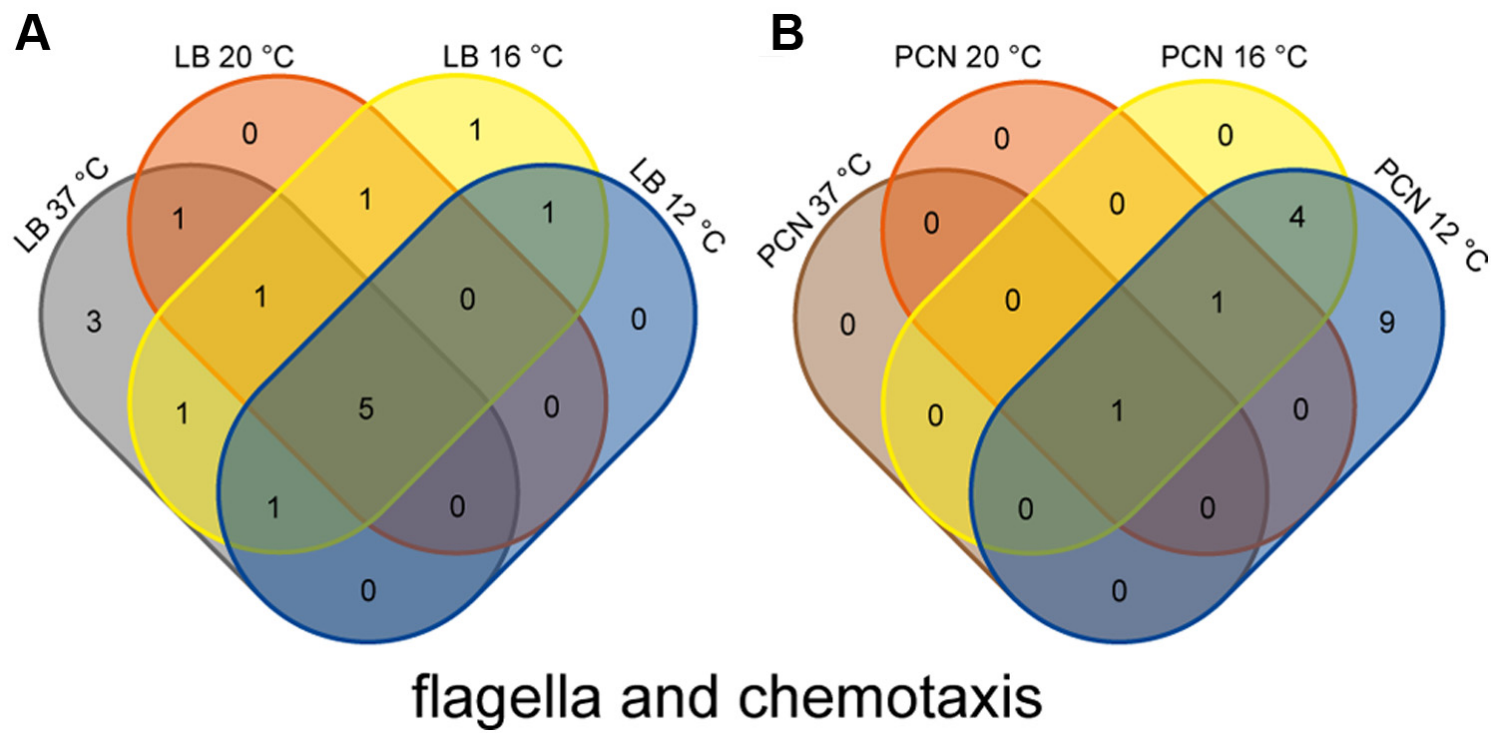
**Differentially abundant chemotaxis and flagellar proteins STM grown in LB or PCN medium at various temperatures.** Proteins were analyzed regarding their occurrence in the compared groups. Proteomic data were subsequently analyzed by comparison to gene ontology groups (34, 35), as shown here for number of flagella and chemotaxis associated proteins in Venn diagrams (*A*, LB; *B*, PCN; supplemental Table S10). Color coding and statistical analyses as described for Figure 1. STM, *Salmonella enterica* serovar Typhimurium; LB, lysogeny broth.

Flagellar- and chemotaxis-associated proteins are detected in STM grown at 37 °C in LB medium. However, not all flagella and chemotaxis-associated proteins could be found in the proteomic data since many of them are membrane proteins. With decrease of temperature (20 °C – 8 °C), we found higher numbers of proteins being uniquely present in 37 °C cultures or being higher abundant in 37 °C cultures (Fig. 5). These observations are in line with a previous finding of expression of flagella genes during log phase. Saini *et al*. (61) revealed a cross-talk of SPI1 gene expression and flagellar gene expression, leading to successive expression of flagellar genes and SPI1 genes. This revealed coordination of expression of flagellar genes and SPI1 genes during log-phase growth. Here, we observed presence of flagellar proteins and SPI1-encoded proteins in STM grown in LB medium at 37 °C.

**Fig. 6:**
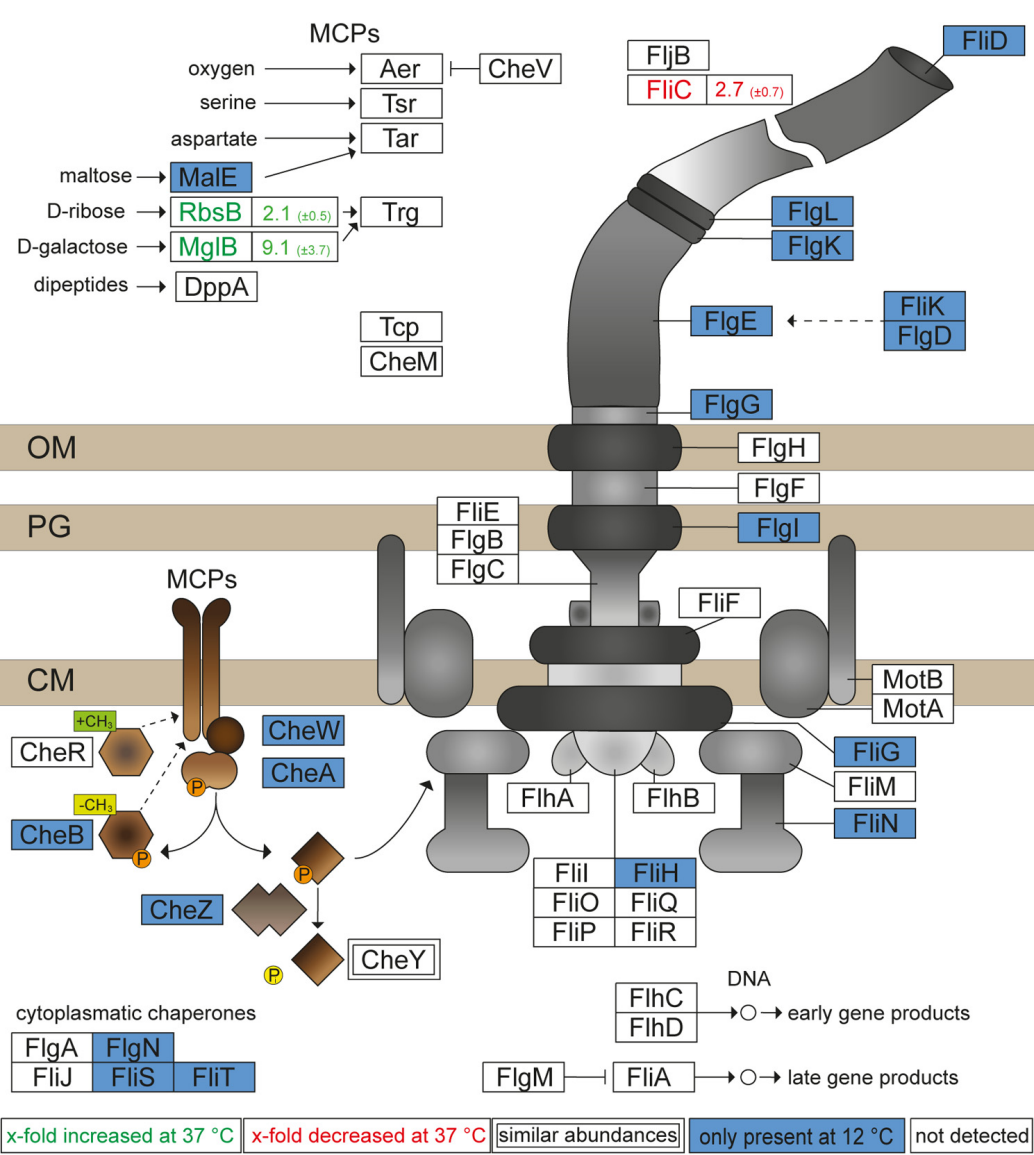
**Growth of STM in PCN medium at 12 °C affects amounts of flagellar and chemotaxis proteins.** Shown are the results of the proteome analysis of STM grown in PCN media at 12 °C compared to 37 °C. Changes in protein abundances are indicated as follows: *blue box*, solely detected in STM grown at 12 °C; *white box*, not detected in STM grown at 37 °C and 12 °C; *green font*, higher abundance in STM grown at 37 °C compared to 12 °C; *red font*, lower abundance in STM grown at 37 °C compared to 12 °C. Figures indicate x-fold change of protein abundance. *Double boxed* proteins indicate similar abundances in both samples. The illustration is based on ‘flagellar assembly’ and ‘bacterial chemotaxis’ of STM by KEGG (79). STM, *Salmonella enterica* serovar Typhimurium.

**Fig. 7:**
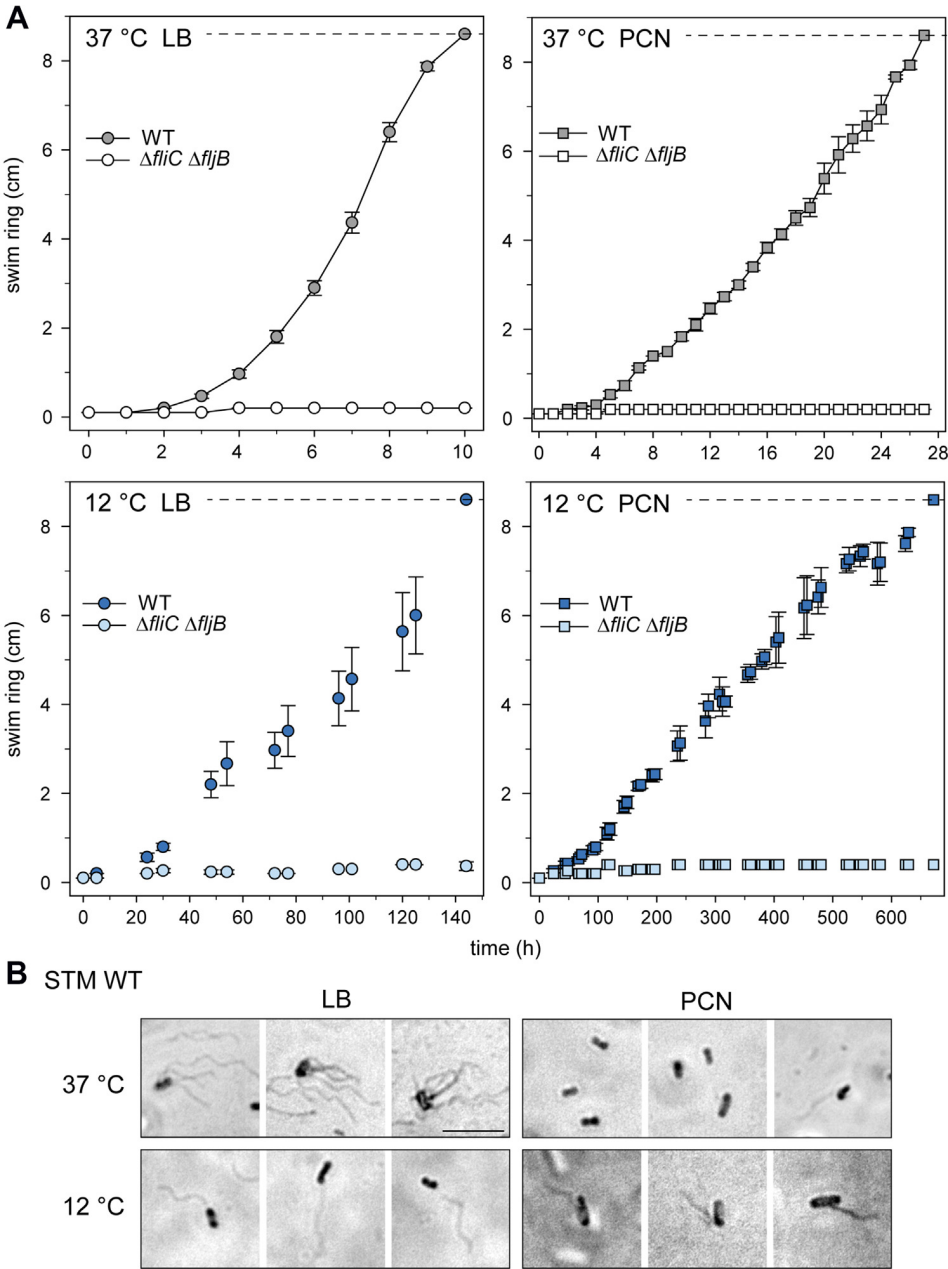
**Swimming motility of STM at 12 °C or 37 °C.***A*, LB or PCN swim agar was inoculated with 1 μl cultures of STM WT or STM Δ*fliC* Δ*fljB* grown according to the respective growth conditions used for proteomic analyses. Plates were incubated at 12 °C or 37 °C and at various times points of culture, swim ring diameters were determined. Images of representative swim zones are shown in supplemental Fig. S3. Shown are means of at least three biological replicates with SD. *B*, visualization of flagella of STM grown in LB and PCN medium at 37 °C and 12 °C by flagella stain. Shown are three randomly selected fields of view of STM grown in LB medium and PCN medium at 37 °C or 12 °C. Images were acquired using a widefield microscope (Zeiss Axio Lab A1) equipped with 100× oil objective and CMOS camera (Bresser MicroCam SP 3.1). The scale bar represents 5 μm. LB, lysogeny broth; STM, *Salmonella enterica* serovar Typhimurium.

STM grown in PCN medium revealed the presence of many flagellar and chemotaxis proteins at a growth temperature of 12 °C. Synthesis of 14 unique proteins and higher abundance of FliC (2.7-fold ±0.73) compared to STM grown in PCN medium at 37 °C was detected. These proteins comprise several flagellar proteins ranging from cytoplasmic chaperones, structural proteins of the basal body, to flagella filament proteins, as well as methyl-accepting chemotaxis proteins, and proteins involved in transduction of chemotactic signals (Fig. 6). These proteins lead to a potentially functional flagella-mediated motility in STM grown in PCN medium at 12 °C. Analyses of STM grown in PCN medium at 37 °C, 20 °C, and 8 °C revealed the absence of flagellar and chemotaxis proteins or rather low abundances. For STM grown at 16 °C in PCN medium, some proteins associated with the flagella were detected. These proteins comprise FliN, FliK, FliD, FlgE, FlgD, and CheA, which might indicate flagella synthesis in PCN medium at lower temperatures (≤16 °C) resulting in the presence of many motility-associated proteins in STM grown at 12 °C but not at 8 °C. However, a study by Blair *et al*. (52) revealed by real-time PCR decreased expression of several genes involved in motility after growth in MOPS minimal media with glucose at 37 °C in STM. These findings are in line with our proteomic analyses. Further, these observations can be explained by presence of glucose in PCN media. Glucose functions as a repressor of flagella biosynthesis due to a cAMP-cAMP receptor protein (cAMP-CRP) binding site in front of the flagellar master regulator operon *flhDC* (62). Whenever glucose is present in the bacterium, the adenylate cyclase is inactive and no cAMP is generated, repressing the flagellar master regulator operon *flhDC*. In the absence of glucose, the adenylate cyclase is active and cAMP is produced, leading to *flhDC* expression and formation of flagella. Nevertheless, flagellar proteins are detected in STM at 12 °C and some flagellar proteins are present at 16 °C in PCN medium with glucose. Therefore, an unknown temperature-dependent regulator might lead to activation of the *flhDC* operon resulting in synthesis of flagella and chemotaxis proteins. To analyze whether the detected flagellar proteins in STM grown at 12 °C in PCN medium led to functional flagella, we performed swim agar experiments and flagella stain to detect flagellar filaments. Swim agar was inoculated with subcultures of STM WT grown in LB or PCN medium at 12 °C, or for comparison at 37 °C, according to the previous used culture conditions. STM WT was able to swim in LB and PCN swim agar at 12 °C and 37 °C (Fig. 7, representative images of swim agar are shown in supplemental Fig. S4). As control, STM Δ*fliC* Δ*fljB* was used lacking the flagella filament. STM WT in LB swim agar at 37 °C reached maximum swim ring extension (8.6 cm diameter) within 10 h (Fig. 7*A*), whereas STM WT in PCN swim agar at 37 °C required 28 h to reach maximal migration (Fig. 7*B*). Motility of STM WT in swim agar at 12 °C was reduced, with a maximal centripetal migration in LB or PCN swim agar at 140 h or 650 h after inoculation, respectively. These observations are in line with the growth kinetics in liquid LB or PCN medium (supplemental Fig. S1) showing that medium composition and growth temperature affect growth rate as well as motility. Control strain STM Δ*fliC* Δ*fljB* did not show formation of swim rings, solely cell mass at inoculation sites was observed with diameters ranging from 0.1 cm to 0.3 cm. Nevertheless, LB and PCN swim agar revealed that under the various conditions applied, STM WT is able to respond chemotactically by flagella-mediated motility.

Next, flagella expressed under the various conditions were visualized using flagella stain (25). STM WT grown in LB at 37 °C possessed multiple flagella per bacterium with typical peritrichous distribution, whereas only few flagellated cells were detected after growth in PCN medium at 37 °C (Fig. 7*B*). We suggest that these STM WT with flagella which are less in number are the only bacteria of the population being able to reach further nutrients and thus be selected for further swimming and growth on swim agar plates resulting in a visible swim ring. STM WT grown at 12 °C expressed only one flagellum per cell which varies in length depending on the medium. Culture of STM WT in LB resulted in cells with one flagellum with length comparable to flagella found on STM WT grown in LB at 37 °C. Flagella on STM WT grown in PCN at 12 °C were shorter in length. Moreover, flagella of STM in 12 °C cultures appeared less intensely stained compared to 37 °C. Control strain Δ*fliC* Δ*fljB* did not show any flagella on the bacterial surface (supplemental Fig. S5).

In summary, flagellar proteins found by proteomic analyses at 12 °C assemble functional flagella mediating motility. Therefore, we suggest that flagella of STM are relevant for the environmental lifestyle outside mammalian hosts. Other studies revealed, for example, the importance of flagella for biofilm formation (63, 64), which occurs often at lower temperatures outside mammalian hosts. An emerging field of research is the role of crop plants as alternative route for propagation human pathogens, the mechanisms deployed by *Salmonella* to colonize fresh produce. Studies demonstrated the importance of directed motility for internalization in lettuce leaves by STM (65) as well as a decreased adhesion to basil leaves for a flagella mutant of *S. enterica* serovar Senftenberg (66) and a decreased adhesion to corn salad leaves for a STM flagella mutant (67).

### Temperature-Dependent Abundances of Virulence-Associated Proteins

**Fig. 8:**
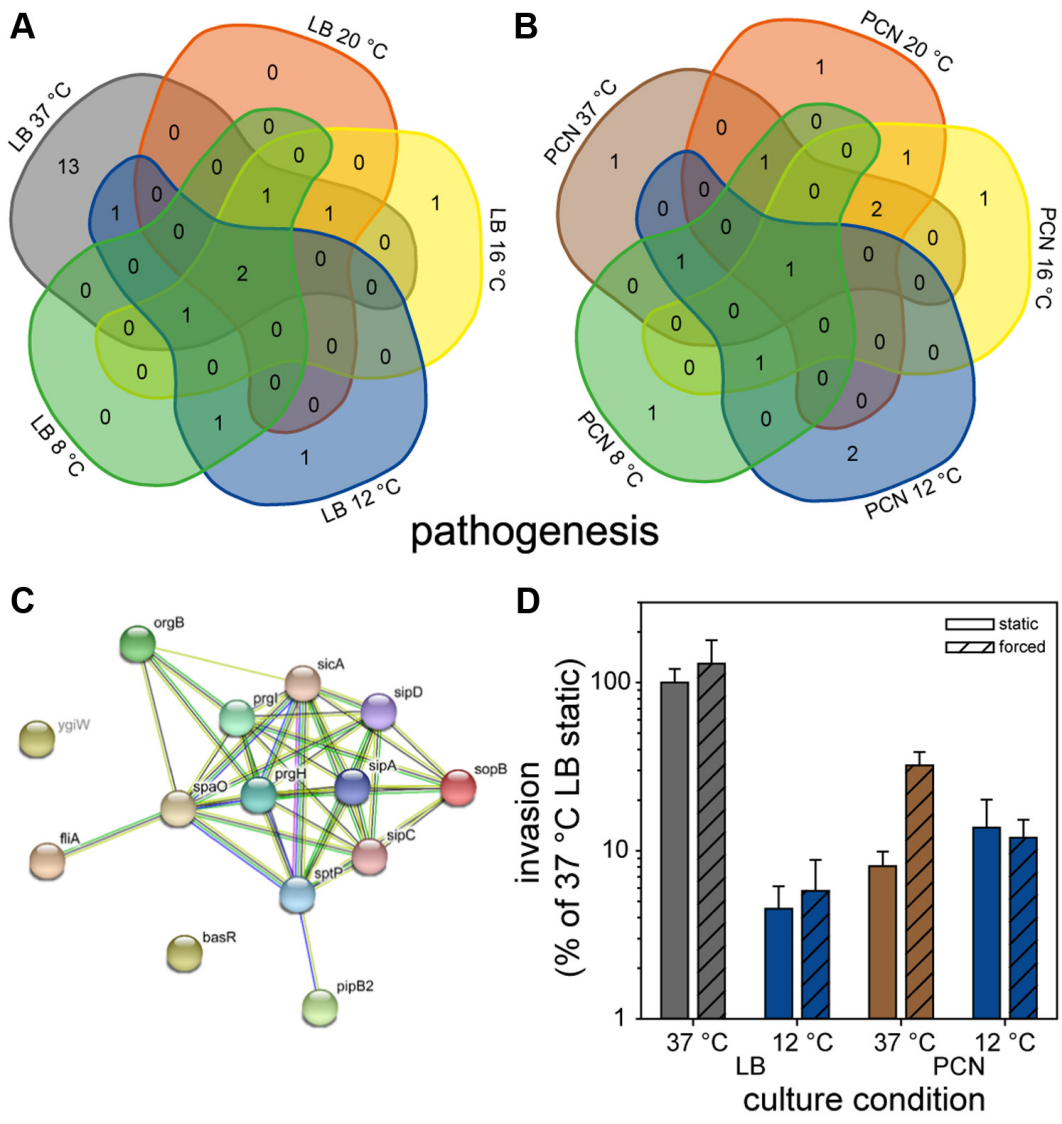
**Differentially abundant proteins involved in pathogenesis in STM grown in LB or PCN media at various temperatures.** Proteins were analyzed regarding their occurrence in the compared groups. Proteomic data were subsequently analyzed by comparison to gene ontology groups (34, 35), as shown here for number of pathogenesis-associated proteins in Venn diagrams (*A*, LB; *B*, PCN; supplemental Table S10). Color coding and statistical analyses as described for Figure 1. *C*, overview of proteins with increased abundance and unique presence in STM grown at 37 °C in LB media compared to STM grown at 8 °C in LB media reveals gene ontology of pathogenesis-related proteins illustrated by STRING (80). Proteins in *gray* font indicate proteins not covered by STRING of *Salmonella* proteome. *D*, invasion of polarized epithelial MDCK epithelial cells by STM WT after culture in LB or PCN at 37 °C or 12 °C. The bacterial inoculum was added to cells without (static) or with (forced) centrifugation to increase contact between STM and MDCK cells. Invasion is expressed as percentage of invasion of STM WT cultured in LB at 37 °C. LB, lysogeny broth; STM, *Salmonella enterica* serovar Typhimurium.

In addition to flagella and chemotaxis proteins, a temperature-dependent regulation of virulence-associated proteins in STM which are commonly associated with pathogenesis in mammalian hosts was observed. Sorting of all STM genes according to gene ontology revealed a group of 83 proteins associated with *Salmonella* pathogenesis. Only three pathogenesis-related proteins were found in STM grown at temperatures lower than 37 °C in LB medium, whereas up to 19 solely or higher abundant pathogenesis-related proteins were found in STM grown at 37 °C in LB medium in comparison to STM grown at lower temperatures (<37 °C) (Fig. 8*A*). STRING networks demonstrate high interconnections of pathogenesis-related proteins found for STM grown in LB at 37 °C being related to SPI1-T3SS (PrgI, PrgH, SpaO), associated proteins (BasR, OrgB, SicA, and YgiW), and effector proteins (SipA, SipC, SipD, SopB, SptP), as well as the third class flagella gene regulator FliA (Fig. 8*C*). In addition, one SPI2-T3SS effector protein, PipB2, was uniquely present in STM grown at 37 °C in LB medium. The giant adhesin SiiE (encoded with its cognate T1SS by SPI4 genes), involved in the coordinated adhesion and invasion process of epithelial cells with SPI1-encoded proteins, was only observed in STM grown in LB medium at 37 °C. Growth of STM in nutrient-rich medium at 37 °C in late–log phase is known as inducing condition for expression of SPI1/SPI4 genes (31, 68, 69). Moreover, no adhesive structures other than SiiE were found for STM grown in LB or PCN medium at 8 °C to 37 °C. These findings are in line with known inducing conditions for expression of genes encoding for adhesive structures, that is, T1SS-secreted adhesin BapA being involved in the formation of biofilms at the air-liquid interface. Expression of *bapA* is co-coordinated with Curli fimbriae and cellulose under static growth conditions at 30 °C (70).

In contrast to growth in LB medium, pathogenesis-related proteins were found in STM grown in PCN medium also at lower temperatures (Fig. 8*B*). In PCN medium, less pathogenesis-related proteins were found in total, but interestingly, PrgI and SipC were present in comparable amounts in STM grown at 37 °C, 20 °C, and 16 °C in PCN medium. Both proteins were not found in STM grown at 12 °C and 8 °C.

Nevertheless, proteins related to pathogenesis were more abundant in STM grown at 37 °C in LB medium, that is, PrgI (29.4-fold ±20.5) and SipC (8.5-fold ±5.4) or only present in LB medium compared to STM grown at 37 °C in PCN medium (supplemental Fig. S2). A previous study investigated the activity of promoter P_*prgH*_ in STM in M9 minimal media with glucose by flow cytometry using a fluorescent reporter. No expression of SPI1 genes was detected in STM grown in M9 minimal media (68). In addition, promoter activity was increased by changing the C-source to saccharate and increasing NaCl to 0.5%. We did not detect PrgH in STM grown in PCN medium, potentially because of the absence of saccharate and NaCl in the PCN medium. Nevertheless, we detected a subset of SPI1-encoded associated proteins even in PCN media at temperatures ranging from 16 °C to 37 °C even in the absence of saccharate and NaCl. However, SPI1-encoded associated proteins are still less abundant compared to amounts of SPI1-encoded associated proteins found in STM grown in LB media.

Furthermore, proteins encoded by genes of the SsrAB regulon, except PipB2 and SseA, were not observed in STM grown in LB or PCN medium at any temperature. Expression of SPI2 genes is known to only occur under nutritional limitations, including Mg^2+^ deprivation, phosphate starvation, and acidic pH, considered to mimic environmental conditions inside the SCV (13, 52). The PCN medium used in this study mimics only nutritional limitations potentially found in the environment but no restrictions in Mg^2+^ and phosphate availability or changes in osmolarity.

In addition to the medium, temperature influences the abundance of pathogenesis-related proteins resulting in no or less pathogenesis-related proteins at lower temperatures. One factor being involved in temperature-dependent expression of pathogenesis genes might be the cold shock-associated exoribonuclease polynucleotide phosphorylase Pnp encoded by *pnp*. Pnp is involved in the adaptation to growth at low temperatures by specifically degrading mRNAs of cold shock proteins, allowing the bacterial replication after shifting the temperature. However, deletion of *pnp* resulted in increased expression of 87 STM genes associated with SPI1 and SPI2. Deletion of *pnp* even led to increased invasion of MDCK cells by STM and increased replication in murine macrophage-like J774A.1 cells and in mouse spleen (71). These results indicate a temperature-dependent regulation of SPI1- and SPI2-encoded proteins (50, 71). Here, we observed increased abundance of Pnp in STM grown in LB medium under lower temperatures (≤16 °C). STM grown at 16 °C in LB medium showed 13.7-fold (±3.1)–increased amount of Pnp compared to 37 °C, whereas differentially abundance decreased with further decrease of growth temperature (12 °C: 9.4-fold ±1.8; 8 °C: 7.0-fold ±1.3). In PCN medium, the amount of Pnp is higher at lower temperatures than STM grown at 37 °C (20 °C: 27.1-fold ±14.8; 16 °C: 23.8-fold ±7.3; 12 °C: 25.4-fold ±7.6; 8 °C: 22.1-fold ±11.89) which might be reasoned by double-stressed STM cells due to nutritional limitations and cold stress. However, increased abundances of Pnp can be in part correlated with the presence of SPI1- and SPI2-encoded associated proteins further depending on temperature. Nevertheless, further investigation is needed.

We determined the effect of growth temperature on invasion of nonphagocytic host cells by STM, a key virulence trait (Fig. 8*D*). Polarized MDCK epithelial cells were used as host cells for invasion by STM WT grown under various culture conditions. Invasion by STM cultured in LB at 12 °C was highly reduced compared to LB culture at 37 °C. STM precultured in PCN also invaded about 9 to 10-fold lower than STM from LB cultures grown at 37 °C. We also forced contact between STM and MDCK cells by centrifugation, to compensate reduced motility. Centrifugation strongly increased invasion of STM cultured in PCN at 37 °C. This indicates that invasion factors encoded by SPI1 and SPI4 genes were expressed under these conditions, yet host cell contact was limited by reduced flagella expression.

Our data indicate a clear SPI1 virulence-associated phenotype for STM only grown in LB medium at higher temperatures and reveal temperature- and medium-dependent presence of pathogenesis-related proteins.

### Single Cell Analyses of Expression of Virulence or Flagella Genes During Growth at 37 °C or 12 °C

**Fig. 9:**
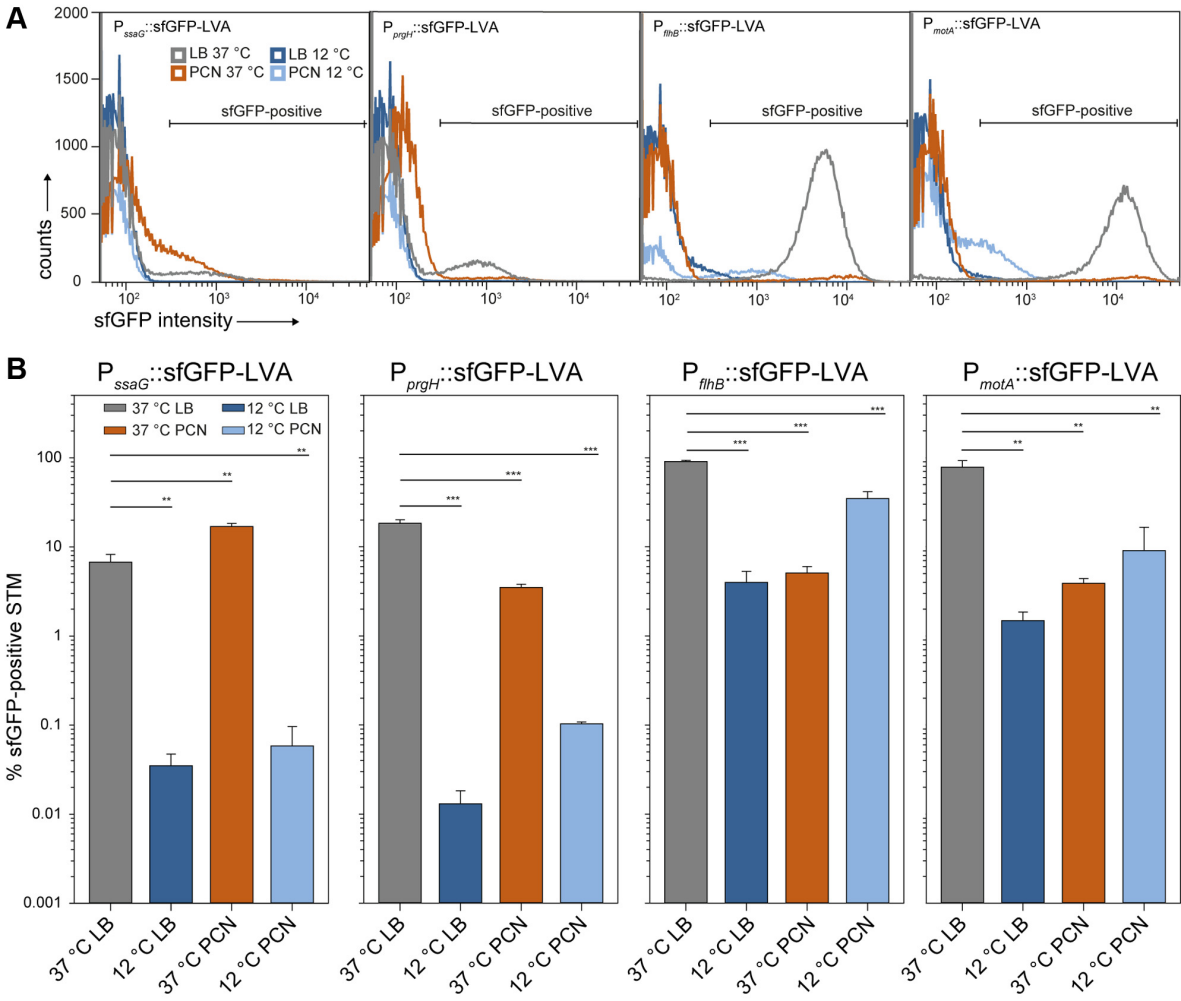
**Single cell analyses of expression of virulence and motility genes in LB or PCN at 37 °C or 12 °C.** STM strains harboring dual fluorescence reporter plasmids. Fluorescence reporters for expression of genes in SPI2 (P_*ssaG*_), SPI1 (P_*prgH*_), flagella class II (P_*flhB*_), or flagella class III (P_*motA*_) were generated as promoter fusions to sfGFP destabilized by the LVA-tag. Reporter plasmids were analyzed in STM WT and culture in LB or PCN was performed at 37 °C or 12 °C as for proteomic analyses. *A*, at least 50,000 bacteria-sized particles were analyzed by flow cytometry and sfGFP fluorescence was determined. Gating of sfGFP-positive STM was performed as described in supplemental Fig. S6. *B*, percentage of sfGFP-positive STM after growth in LB or PCN at 37 °C or 12 °C. Mean percentages and SD of three biological replicates with four technical replicates are shown. Statistical analysis was performed by one-way ANOVA and significance is indicated as: ∗∗*p* < 0.01; ∗∗∗*p* < 0.001. LB, lysogeny broth; STM, *Salmonella enterica* serovar Typhimurium; SPI, Salmonella pathogenicity island.

While proteomic analyses reflect alterations of protein amounts in the entire STM population in response to environmental cues, the response of individual STM cells may be highly divergent. For example, expression of genes in SPI1 and resulting invasiveness is restricted to a subpopulation of STM subcultured at 37 °C in rich media (72, 73). Here, we applied previously established fluorescent protein reporters (26, 74) for single cell analyses of activation of P_*ssaG*_, P*_prgH_*, P*_flhB_*, or P*_motA_* as promoters representing the regulons of SPI2-T3SS, SPI1-T3SS, flagella class II, or flagella class III genes, respectively. The proper performance of the reporters was confirmed by analyses of the background of STM WT or strains defective in the cognate regulatory system (supplemental Fig. S6). Single cell analyses were performed by flow cytometry for STM WT harboring various reporter plasmids after growth at 37 °C or 12 °C in LB or PCN media under conditions identical to the proteomic analyses (Fig. 9).

The percentage of STM expressing the P*_ssaG_*::sfGFP fusion and levels of expression were low under all conditions, in line with prior observations that the regulon is activated by P_i_ limitation and acid pH (13, 75). After culture at 37 °C, 8.7% ±0.2 or 17.9% ±0.5 positive STM were determined in LB or PCN cultures, respectively, while percentage of P*_ssaG_*::sfGFP-induced STM after 12 °C culture was below 0.2%.

For SPI1 promoter P*_prgH_*, culture at 37 °C resulted in 16.2% ±0.5 or 3.9% ±0.1 induced STM in LB or PCN cultures respectively, and higher expression levels in LB cultures at 37 °C are in line with induction of invasiveness under these culture conditions. Induction in 12 °C cultures was highly reduced. These expression patterns were reflected by proteomic identification of SPI1-T3SS subunits and effector proteins (Fig. 8*C*).

Both reporters for activation of class II or class III flagella genes were highly induced in 37 °C LB cultures, with 87.6% ±0.4 or 64.9% ±0.7 for P_*flhB*_ or P_*motA*_ promoters, respectively, matching prior observations (61). Also, expression levels of these reporters were highest among the fusions analyzed. In LB culture at 12 °C and PCN culture at 37 °C, expression levels and proportion of expressing STM were highly reduced. Interestingly, in cultures grown in PCN at 12 °C, 39.3% ±4.3 or 19.7% ±1.2 of STM were positive for induction of P_*flhB*_::sfGFP or P_*motA*_::sfGFP fusions, respectively, however sfGFP levels of positive cells were highly reduced compared to STM grown in LB at 37 °C.

The expression of flagella genes in PCN cultures grown at 12 °C is in line with the proteomic identification, detection of flagella filaments, and motility. Single cell data indicate that a lower number of STM cells in PCN at 12 °C can express and assemble functional flagella, despite presence of glucose that repressed flagella expression at 37 °C.

## Conclusions

The present study demonstrates the effect of growth temperature and medium in proteome composition of STM. We showed an increase of metabolic proteins in STM grown at lower temperatures, and additionally in minimal medium. Further, we observed the activation of cold stress response in STM grown at lower temperatures, whereas proteins related to heat stress were found in higher abundances in STM grown at 37 °C. Additionally, proteins associated to pathogenesis and being involved in adhesion to and invasion of epithelial cells were found in large amounts only in STM grown in rich medium at 37 °C. Flagellar and chemotaxis proteins were found in high abundance in STM grown in LB medium at 37 °C but not or only in less abundance in STM grown at lower temperatures and in STM grown in PCN medium (37 °C, 20 °C, 8 °C). Interestingly, we observed several flagellar proteins ranging from cytoplasmic chaperones, structural proteins of the basal body to flagella filament proteins, as well as methyl-accepting chemotaxis proteins and proteins involved in transduction of chemotactic signals in STM grown in PCN medium at 12 °C. In further experiments, we demonstrated flagella synthesis and assembly at 12 °C in PCN and LB medium leading to motile bacteria. Further experiments are required to elucidate the potential impact on virulence for STM under various environmental conditions and for emerging propagation routes such as crop plants. In regard to temperatures ranging from 12 °C to 16 °C for cultivation of plants under field conditions and potential nutritional limitations in soil and on plants, the results further imply potential SPI1-T3SS–independent colonization and invasion of plants. SPI1- and SPI4-encoded proteins are of crucial importance for adhesion and invasion of epithelial cells present in mammalian hosts and therefore potentially only synthesized under conditions mimicking the nutrient-rich environment at host body temperatures. Yet, our data indicate low level expression of SPI1 genes and invasiveness of STM after culture in minimal media at 12 °C. In addition, the synthesis of flagella at lower temperatures in minimal medium potentially further contributes to the colonization of plants. This hypothesis is further supported by studies revealing the involvement of flagella-mediated motility in adhesion to several plants (67, 76, 77, 78).

In summary, we observed changes in the proteome of STM depending on changes in growth medium and temperature with potential influences on STM virulence in various environments (mammalian hosts *versus* plants).

## Data Availability

The mass spectrometry proteomics data have been deposited to the ProteomeXchange Consortium via the PRIDE partner repository with the dataset identifier PXD025848.

## Supplemental data

This article contains supplemental data (18, 19, 26, 81).

## Conflict of interest

The authors declare that they have no conflicts of interest with the contents of this article.

## Supplemental Data

Suppl. Figure 1

Suppl. Figure 2

Suppl. Figure 3

Suppl. Figure 4

Suppl. Figure 5

Suppl. Figure 6

Suppl. Table 1

Suppl. Table 2

Suppl. Table 3

Suppl. Table 4

Suppl. Table 5

Suppl. Table 6

Suppl. Table 7

Suppl. Table 8

Suppl. Table 9

Suppl. Table 10
