# Supplementary material for: Effect of Environmental Temperatures on Proteome Composition of Salmonella enterica Serovar Typhimurium
Source: Mol Cell Proteomics. 2022 Jul 2;21(8):100265. doi: 10.1016/j.mcpro.2022.100265 (PMC9396072; doi:10.1016/j.mcpro.2022.100265)
Supplement: Suppl. Figure 1 [file mmc1.pdf]

Supplementary Material to 'Effect of environmental temperatures on proteome composition of *Salmonella enterica* serovar Typhimurium'

Laura Elpers, Jörg Deiwick, Michael Hensel

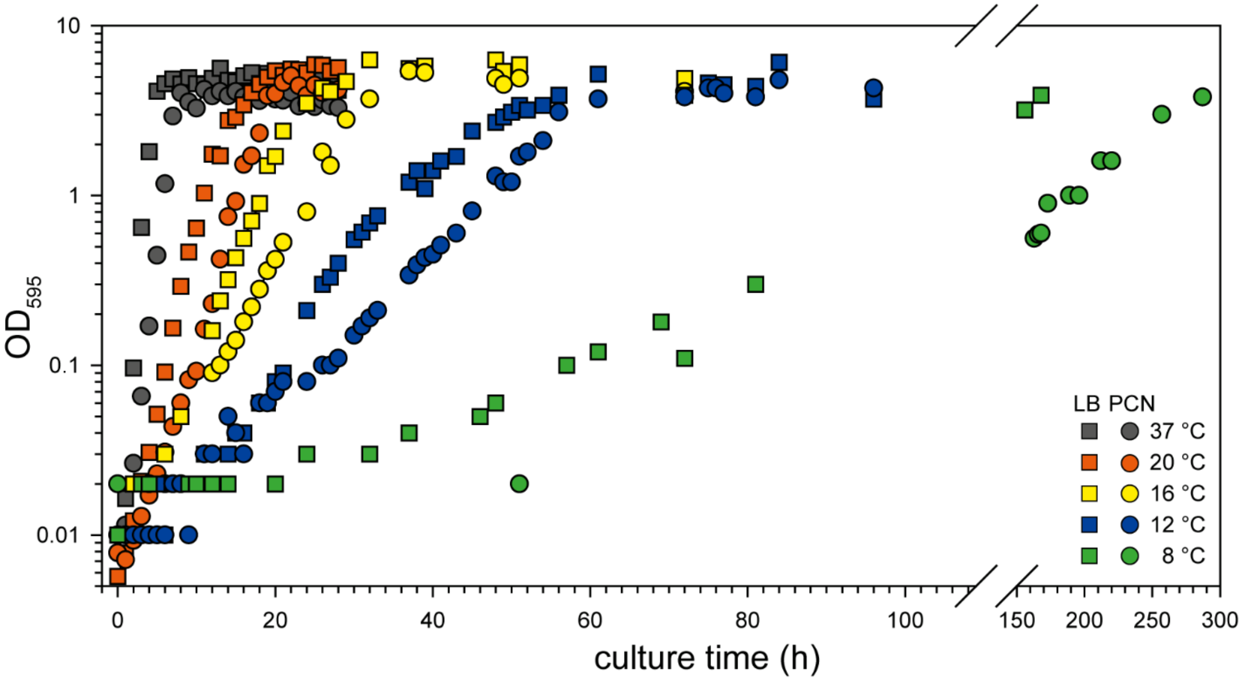

Supplementary Figure 1: Growth kinetics of STM WT in LB or PCN medium at 8 °C, 12 °C, 16 °C, 20 °C, or 37 °C. STM WT were grown o/n at 37 °C in LB or PCN medium, and cultures were used to inoculate 100 ml fresh LB or PCN media to an OD_{595} of 0.01. OD_{595} was determined in intervals until no further increase in OD_{595} was observed.
