# Supplementary material for: Effect of Environmental Temperatures on Proteome Composition of Salmonella enterica Serovar Typhimurium
Source: Mol Cell Proteomics. 2022 Jul 2;21(8):100265. doi: 10.1016/j.mcpro.2022.100265 (PMC9396072; doi:10.1016/j.mcpro.2022.100265)
Supplement: Suppl. Figure 2 [file mmc2.pdf]

Supplementary Material to 'Effect of environmental temperatures on proteome composition of *Salmonella enterica* serovar Typhimurium'

Laura Elpers, Jörg Deiwick, Michael Hensel

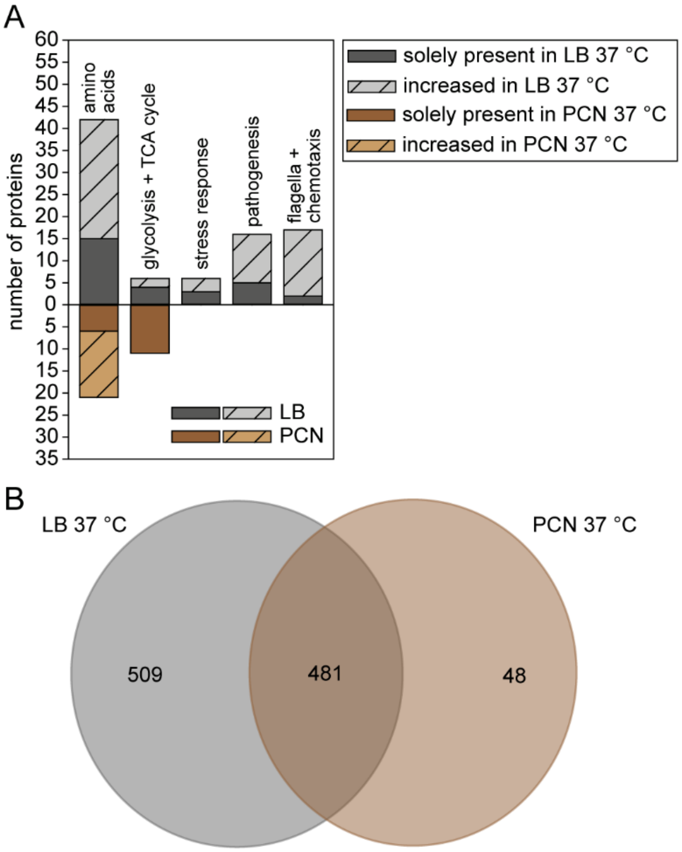

Supplementary Figure 1: Differentially abundant proteins of various ontology groups in STM grown in LB or PCN medium at 37 °C. (A) Proteomic data were subsequently analyzed by comparison to gene ontology groups, as shown here for distributions of numbers of proteins classified according to protein classes 'amino acids' (= proteins involved in amino acid metabolism), 'glycolysis + TCA cycle', 'stress response', 'pathogenesis', and 'flagella and chemotaxis'. Grey plain bars indicate higher abundances of proteins in STM grown in LB media, whereas brown plain bars represent lower abundances of proteins grown in LB media in comparison to PCN media. Further, hatched bars indicate the presence of numbers of proteins solely found in one group, in STM grown in LB (hatched grey) or PCN media (brown colored). Proteins were analyzed with regard to their occurrence in the compared groups and depicted in a Venn diagram (B). Statistical analyses were performed as described for **Figure 1**.
