# Supplementary material for: Effect of Environmental Temperatures on Proteome Composition of Salmonella enterica Serovar Typhimurium
Source: Mol Cell Proteomics. 2022 Jul 2;21(8):100265. doi: 10.1016/j.mcpro.2022.100265 (PMC9396072; doi:10.1016/j.mcpro.2022.100265)
Supplement: Suppl. Figure 3 [file mmc3.pdf]

Supplementary Material to 'Effect of environmental temperatures on proteome composition of *Salmonella enterica* serovar Typhimurium'

Laura Elpers, Jörg Deiwick, Michael Hensel

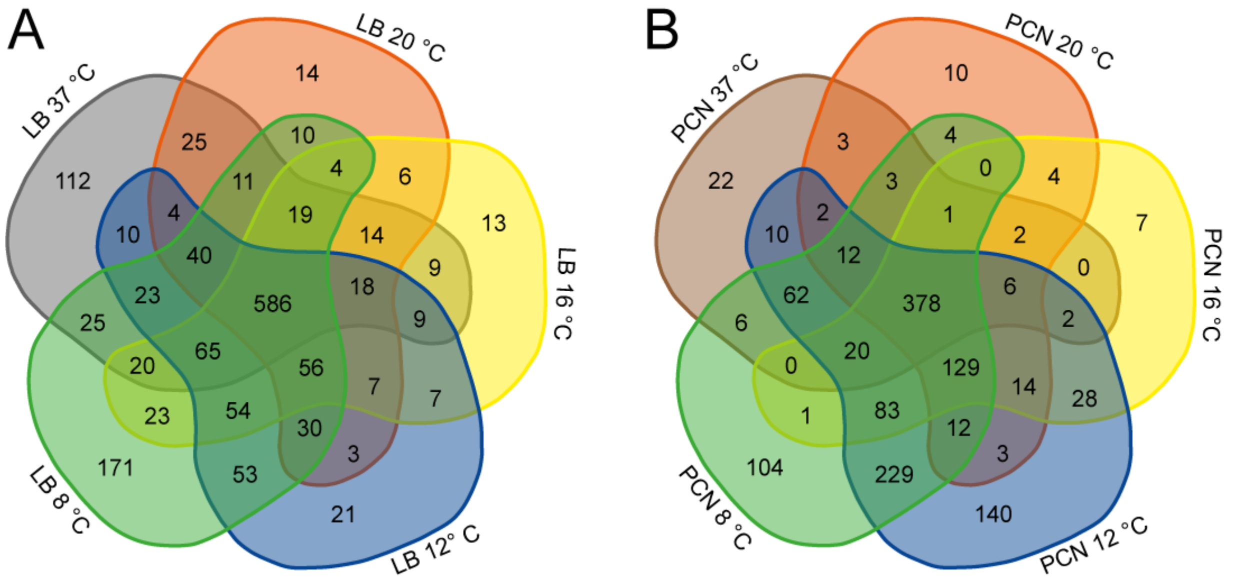

Supplementary Figure 1: Differentially abundant proteins of STM grown in LB or PCN medium at various temperatures. Total proteins were analyzed and compared to each temperature used for the respective medium (**A**, **B**), results are shown in Venn diagrams. Color coding and statistical analyses as described for **Figure 1**.
