# Supplementary material for: Effect of Environmental Temperatures on Proteome Composition of Salmonella enterica Serovar Typhimurium
Source: Mol Cell Proteomics. 2022 Jul 2;21(8):100265. doi: 10.1016/j.mcpro.2022.100265 (PMC9396072; doi:10.1016/j.mcpro.2022.100265)
Supplement: Suppl. Figure 4 [file mmc4.pdf]

Supplementary Material to ‘Effect of environmental temperatures on proteome composition of *Salmonella enterica* serovar Typhimurium’

Laura Elpers, Jörg Deiwick, Michael Hensel

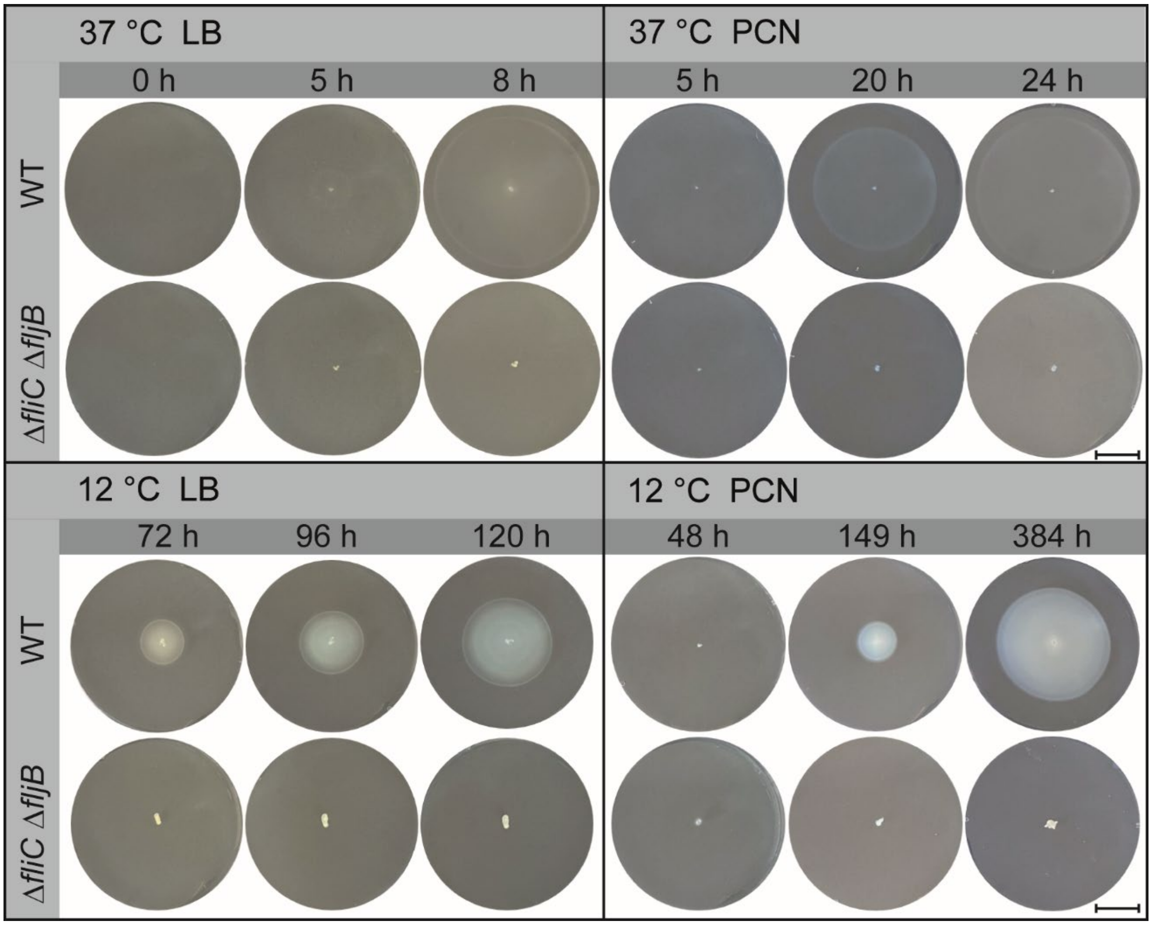

Supplementary Figure 1: Swimming motility of STM WT and STM Δ*fliC* Δ*fljB* in LB swim agar or PCN swim agar at 12 °C or 37 °C. Representative images of swim agar plates at indicated time points after central inoculation are shown. Scale bar, 2 cm.
