# Supplementary material for: Effect of Environmental Temperatures on Proteome Composition of Salmonella enterica Serovar Typhimurium
Source: Mol Cell Proteomics. 2022 Jul 2;21(8):100265. doi: 10.1016/j.mcpro.2022.100265 (PMC9396072; doi:10.1016/j.mcpro.2022.100265)
Supplement: Suppl. Figure 5 [file mmc5.pdf]

Supplementary Material to ‘Effect of environmental temperatures on proteome composition of *Salmonella enterica* serovar Typhimurium’

Laura Elpers, Jörg Deiwick, Michael Hensel

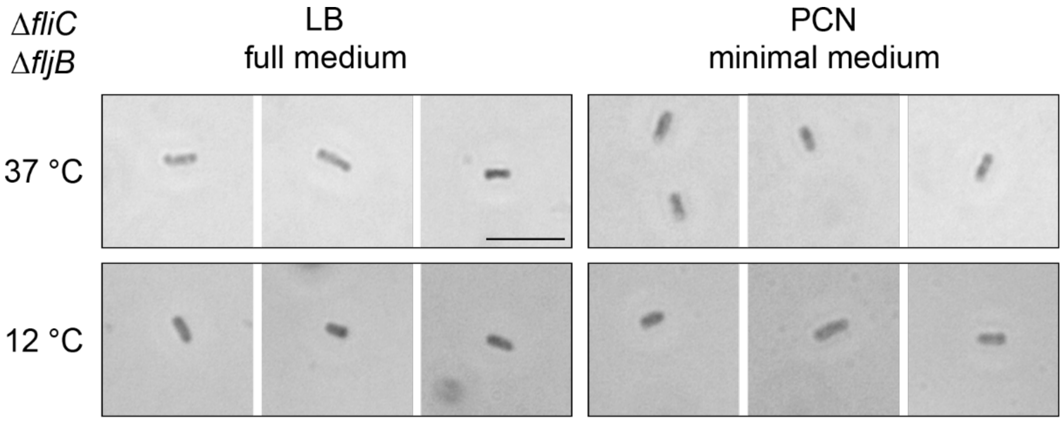

Supplementary Figure 1: Flagella stain for visualization of flagella of STM Δ*fliC* Δ*fljB* grown in LB or PCN medium at 37 °C or 12 °C. Shown are three randomly selected images of STM Δ*fliC* Δ*fljB* grown in LB medium or PCN medium at 37 °C or 12 °C. Image acquisition as for **Figure 7**. Scale bar, 5 µm.
