# Supplementary material for: Effect of Environmental Temperatures on Proteome Composition of Salmonella enterica Serovar Typhimurium
Source: Mol Cell Proteomics. 2022 Jul 2;21(8):100265. doi: 10.1016/j.mcpro.2022.100265 (PMC9396072; doi:10.1016/j.mcpro.2022.100265)
Supplement: Suppl. Figure — 6 [file mmc6.pdf]

Supplementary Material to ‘Effect of environmental temperatures on proteome composition of *Salmonella enterica* serovar Typhimurium’

Laura Elpers, Jörg Deiwick, Michael Hensel

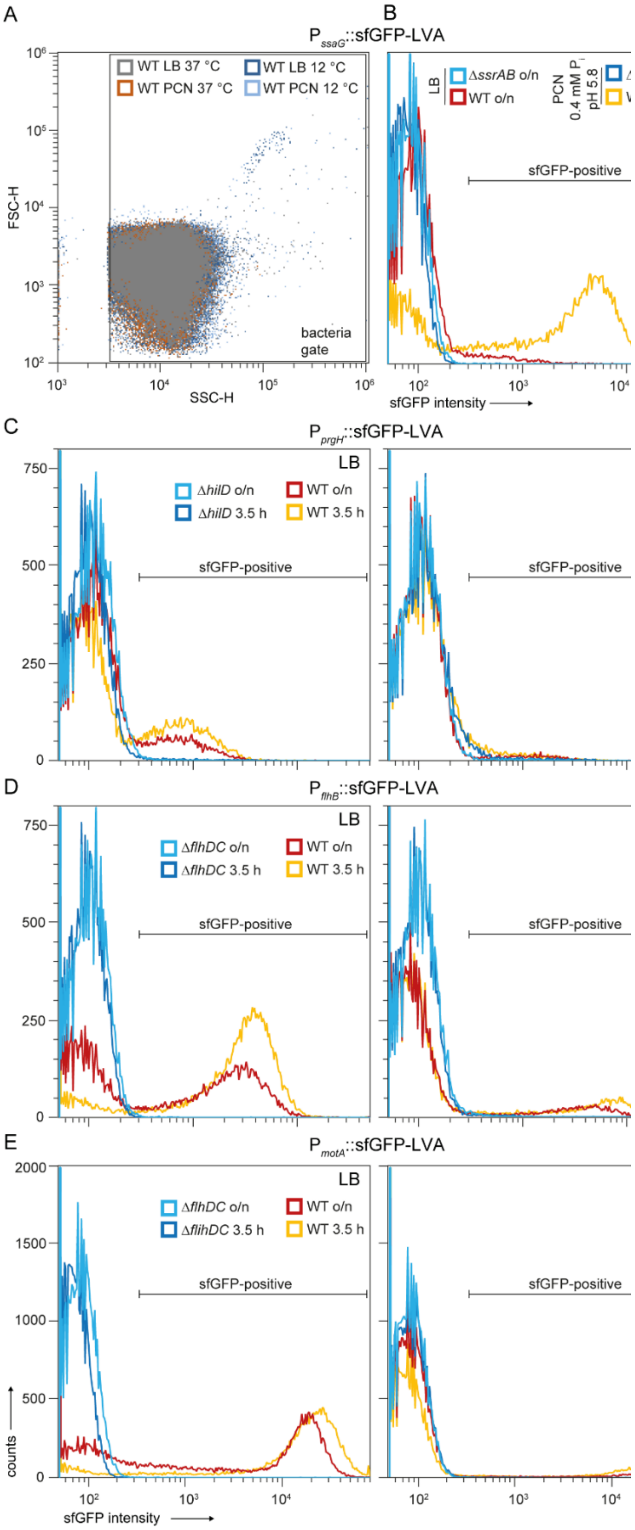

Supplementary Figure S: Gating strategy for flow cytometry and controls for reporter response. Fluorescence reporters for expression of genes in SPI2 (P_{ssaG}), SPI1 (P_{prgH}), flagella class II (P_{flhB}), or flagella class III (P_{motA}) were generated as promoter fusions to sfGFP destabilized by the LVA-tag. Reporter plasmids were analyzed in STM WT, or mutant strains defective in *ssrAB* (regulators of SPI2 genes), *hilD* (regulator of SPI1 genes), or *flhDC* (regulator of flagella genes) as indicated. After o/n culture in LB or PCN media at 37 °C or 12 °C, cultures were fixed and analyzed by flow cytometry using a Attune NxT instrument. Bacteria-sized particles were defined by SSC/FSC and sfGFP fluorescence of at least 50,000 STM in the bacteria gate was determined (**A**). **B**) The STM WT or Δ*ssrAB* strains harboring the P_{ssaG} reporter were grown at 37 °C in LB or PCN with 0.4 mM inorganic phosphate at pH 5.8 as conditions non-inducing or inducing expression of SPI2 genes, respectively. **C, D, E**) STM WT, Δ*hilD* or Δ*flhDC* strains harboring reporter plasmids with P_{prgH}::sfGFP-LVA, P_{flhB} ::sfGFP-LVA, or P_{motA}::sfGFP-LVA as indicated were grown in LB or PCN for 3.5 h or o/n. The gate for sfGFP-positive STM was set to <1% positive STM for the respective regulator mutant strain with reporter plasmid, and the gating was applied for quantification of expression at various growth temperature shown in **Figure 9**.
