# Supplementary material for: Effect of Environmental Temperatures on Proteome Composition of Salmonella enterica Serovar Typhimurium
Source: Mol Cell Proteomics. 2022 Jul 2;21(8):100265. doi: 10.1016/j.mcpro.2022.100265 (PMC9396072; doi:10.1016/j.mcpro.2022.100265)
Supplement: Suppl. Table 1 [file mmc7.pdf]

Supplementary Material to 'Effect of environmental temperatures on proteome composition of *Salmonella enterica* serovar Typhimurium'

Laura Elpers, Jörg Deiwick, Michael Hensel

Supplementary Table 1. Bacterial strains used in this study.

Designation	Relevant characteristics	reference
NCTC 12023	STM WT	Lab collection
MvP372	Δ*ssrAB*::*aph*, Km^r	this study
MvP1760	Δ*fliC*::FRT Δ*fljB*::*aph*, Km^r	(1)
MvP2930	Δ*hilD*::*aph*, Km^r	this study
MvP2931	Δ*flhDC*::*aph*, Km^r	this study
MvP2940	Δ*hilD*::FRT	this study

References:

1. Fulde, M., Sommer, F., Chassaing, B., van Vorst, K., Dupont, A., Hensel, M., Basic, M., Klopfleisch, R., Rosenstiel, P., Bleich, A., Backhed, F., Gewirtz, A. T., and Hornef, M. W. (2018) Neonatal selection by Toll-like receptor 5 influences long-term gut microbiota composition. *Nature* 560, 489-493
