# Supplementary material for: Effect of Environmental Temperatures on Proteome Composition of Salmonella enterica Serovar Typhimurium
Source: Mol Cell Proteomics. 2022 Jul 2;21(8):100265. doi: 10.1016/j.mcpro.2022.100265 (PMC9396072; doi:10.1016/j.mcpro.2022.100265)
Supplement: Suppl. Table 2 [file mmc8.pdf]

Supplementary Material to 'Effect of environmental temperatures on proteome composition of *Salmonella enterica* serovar Typhimurium'

Laura Elpers, Jörg Deiwick, Michael Hensel

Supplementary Table 2. Oligonucleotides used in this study.

Designation	Sequence 5´-3´	Purpose
Vf-p4889	ATGCGCAAAGGCGAAGAACTGTTTACCGGTGTGGTGCCGA	GA
Vr-p4889	GGCCGGCATCACCGGCGCCACAGGTGCGGTTG	GA
1f p4889-PssaG	TGTGGCGCCGGTGATGCCGGCCACCGCGACGGTAATGACTC	GA
1r PssaG-sfGFP-2	GCATATGTATATCTCCTTCTTAAATCTAGAAATGCTTTTCCTTAAAATAAATACATCGT	GA
1f p4889-PprgH	CTGTGGCGCCGGTGATGCCGGCCATGTTATTTTAATGTTCCTTACTGGTATCCTA	GA
1r PprgH-sfGFP-2	GCATATGTATATCTCCTTCTTAAATCTAGAATATACTGTTAGCGATGTCTGTCGTTC	GA
Vf-p4889-RBS	TTTAAGAAGGAGATATACATATGCGCAAAGGCGAAGAACTGTTTACCG	GA
Vr-pMW211	GATCCTCTACGCCGGACG	GA
1f PflhB-p4889	GTCCGGCGTAGAGGATCGCGAGAACGAAAGCTTGCT	GA
1r p4889-PflhB	GCGCATATGTATATCTCCTTCTTAAATGATAAGAGAGAATGATGCCAGAACC	GA
1f PmotA-p4889	GTCCGGCGTAGAGGATCTTACCCATGCGCATCAGCCCGTA	GA
1r p4889-PmotA	GCGCATATGTATATCTCCTTCTTAAAGACTATGACAGGATGCGCAGTCG	GA
p4889-LVA SDM For	CTACGCTTTAGTAGCTTAATAAAAGCTTCTGTTTTGG	SDM
sfGFP-LVA Rev	TTTTCGTCGTTTGCAGCTTTATACAGTTCATCCATGC	SDM
ssrA-red-del	ATCAAGTGCCAAAGATTTTGCAACAGGCAACTGGAGGGAAGCATTGTGTAGGCTGGAGCTGCTTC	λRed
ssrB-red-del	CTCATCAAAATATGACCAATGCTTAATACCATCGGACGCCCCTGGCATATGAATATCCTCCTTA	λRed
hilD-Del13-for	AAAATCAATTTATTCTGTATAATGCGTCTCAACACATATTATTCCGGGGATCCGTCGACC	λRed

Designation	Sequence 5´-3´	Purpose
hilD-Del13-rev	TTAATAAAAATCTTTACTTAAGTGACAGATACAAAAAATGTGTAGGCTGGAGCTGCTTCG	λRed
flhD-Del13-for	GTGCGGCTACGTCGCACAAAAATAAAGTTGGTTATTCTGGATTCCGGGGATCCGTCGACC	λRed
flhC-Del13-rev	ATGACTTACCGCTGCTGGAGTGTTTGTCCACACCGTTTCGTGTAGGCTGGAGCTGCTTCG	λRed
k1-red-del	CAGTCATAGCCGAATAGCCT	colony PCR
ssrA delcheck-for	CTTCCTAATGATAACACCATCG	colony PCR
hilD-delcheck-for	TAGCAGCAGATTACCGCACA	colony PCR
hilD-delcheck-rev	TGCCGGCCTTAATCCACAGG	colony PCR
flhCD-delcheck-for	ATAACGCCAGGATAATAGAT	colony PCR
