# Supplementary material for: Effect of Environmental Temperatures on Proteome Composition of Salmonella enterica Serovar Typhimurium
Source: Mol Cell Proteomics. 2022 Jul 2;21(8):100265. doi: 10.1016/j.mcpro.2022.100265 (PMC9396072; doi:10.1016/j.mcpro.2022.100265)
Supplement: Suppl. Table 3 [file mmc9.pdf]

Supplementary Material to ‘Effect of environmental temperatures on proteome composition of *Salmonella enterica* serovar Typhimurium’

Laura Elpers, Jörg Deiwick, Michael Hensel

Supplementary Table 3. Plasmids used in this study.

Designation	Relevant characteristics	reference
pKD13	*aph* resistance cassette flanked by FRT sites, temp-sensitive replication (30 °C), Km^r $Carb^r$	(1)
pWRG730	Red recombinase expression	(2)
pE-FLP	FLP recombinase expression	(3)
p4889	template vector P_{uhpT}::sfGFP, $Carb^r$	(4)
p5879	P_{ssaG}::sfGFP, $Carb^r$	this study
p5880	P_{prgH}::sfGFP, $Carb^r$	this study
p6019	P_{flhB}::sfGFP, $Carb^r$	this study
p6023	P_{motA}::sfGFP, $Carb^r$	this study
p6046	P_{ssaG}::sfGFP-LVA, $Carb^r$	this study
p6047	P_{prgH}::sfGFP-LVA, $Carb^r$	this study
p6048	P_{flhB}::sfGFP-LVA, $Carb^r$	this study
p6091	P_{motA}::sfGFP-LVA, $Carb^r$	this study

References

1. Datsenko, K. A., and Wanner, B. L. (2000) One-step inactivation of chromosomal genes in *Escherichia coli* K-12 using PCR products. *Proc. Natl. Acad. Sci. U S A* 97, 6640-6645
2. Hoffmann, S., Schmidt, C., Walter, S., Bender, J. K., and Gerlach, R. G. (2017) Scarless deletion of up to seven methyl-accepting chemotaxis genes with an optimized method highlights key function of CheM in *Salmonella* Typhimurium. *Plos One* 12, e0172630
3. St-Pierre, F., Cui, L., Priest, D. G., Endy, D., Dodd, I. B., and Shearwin, K. E. (2013) One-step cloning and chromosomal integration of DNA. *ACS Synth Biol* 2, 537-541
4. Röder, J., and Hensel, M. (2020) Presence of SopE and mode of infection result in increased *Salmonella*-containing vacuole damage and cytosolic release during host cell infection by *Salmonella enterica*. *Cell Microbiol* 22, e13155
