# Supplementary material for: Effect of Environmental Temperatures on Proteome Composition of Salmonella enterica Serovar Typhimurium
Source: Mol Cell Proteomics. 2022 Jul 2;21(8):100265. doi: 10.1016/j.mcpro.2022.100265 (PMC9396072; doi:10.1016/j.mcpro.2022.100265)
Supplement: Suppl. Table 4 [file mmc10.pdf]

Supplementary Material to 'Effect of environmental temperatures on proteome composition of *Salmonella enterica* serovar Typhimurium'

Laura Elpers, Jörg Deiwick, Michael Hensel

**Supplementary Table 1. Reagents used in this study.**

| Reagent | Supplier | Article number |
|---|---|---|
| Bacto Tryptone | BD | #211699 |
| Bacto Yeast extract | BD | #212720 |
| Bacto agar | BD | #214030 |
| Gentamicin | Roth | 0233.2 |
| MOPS | Roth | 6979.3 |
| Tricine | Roth | 6997.3 |
| $FeCl_3$ | Merck | 1.03943 |
| $K_2SO_4$ | Merck | 1.05152 |
| NaCl | Roth | 3957.1 |
| $K_2HPO_4$ | Merck | 1.05101 |
| $KH_2PO_4$ | Merck | 1.05104 |
| Glucose | Merck | 1.08342 |
| $NH_4Cl$ | Merck | 1.01145 |
| $MgSO_4$ | Applichem Pancreac | A-1037 |
| $CaCl_2$ | Roth | 5239.1 |
| $Na_2MoO_4$ x 2 $H_2O$ | Merck | 1.06521 |
| NaSeO3 x 5 $H_2O$ | Sigma-Aldrich | S-5261 |
| $H_3BO_3$ | Roth | 69431 |
| $CoCl_2$ x 6 $H_2O$ | Merck | 1.02539 |
| $CuSO_4$ x 5 $H_2O$ | Merck | 1.02790 |
| $MnCl_2$ x 4 $H_2O$ | Merck | 1.05833 |
| $ZnSO_4$ x 7 $H_2O$ | Merck | 1.0279 |
| Urea | Biomol | #50250 |
| Tris | Biomol | # 08003 |
| HCl | Applichem | #A0659 |

| | | |
|---|---|---|
| DTT | Sigma-Aldrich | #D0632 |
| Reagent | Supplier | Article number |
| ammonium bicarbonate | Fluka | #09830 |
| $NH_3$ | Riedel-de Haën | #05002 |
| Iodoacetamide | Sigma Ultra | #068K5302 |
| TFA | J.T. Baker | #10748191 |
| Acetonitrile | J.T. Baker | #10444621 |
| Formic acid | Merck | #100264 |
| Tannic acid | Serva | #35753 |
| $AlK(SO_4)_2 * 12\ H_2O$ | Sigma-Aldrich | #A7210 |
| Crystal violet | Sigma | #C3886 |
| Formaldehyde | Merck | #1.04003 |
