# Supplementary material for: Effect of Environmental Temperatures on Proteome Composition of Salmonella enterica Serovar Typhimurium
Source: Mol Cell Proteomics. 2022 Jul 2;21(8):100265. doi: 10.1016/j.mcpro.2022.100265 (PMC9396072; doi:10.1016/j.mcpro.2022.100265)
Supplement: Suppl. Table 5 [file mmc11.pdf]

Supplementary Material to ‘Effect of environmental temperatures on proteome composition of *Salmonella enterica* serovar Typhimurium’

Laura Elpers, Jörg Deiwick, Michael Hensel

Supplementary Table 5. Settings for label-free protein quantification by mass spectrometry.

	MS	MS precursor selection	MS/MS
**Resolution**	70,000		17,500
**AGC target**	$3x10^6$	$5x10^2$	$1x10^5$
**Max IT**	50 ms		80 ms
**MS Range**	375-1,800 m/z		
**Loop Count**			10
**NCE**			27
**Isolation Width**			1.4 m/z
**charge**		2-5	
