# Supplementary material for: Effect of Environmental Temperatures on Proteome Composition of Salmonella enterica Serovar Typhimurium
Source: Mol Cell Proteomics. 2022 Jul 2;21(8):100265. doi: 10.1016/j.mcpro.2022.100265 (PMC9396072; doi:10.1016/j.mcpro.2022.100265)
Supplement: Suppl. Table 6 [file mmc12.pdf]

Supplementary Material to ‘Effect of environmental temperatures on proteome composition of *Salmonella enterica* serovar Typhimurium’

Laura Elpers, Jörg Deiwick, Michael Hensel

Supplementary Table 1. Generation times in min. of STM WT growth in LB or PCN media at 8 °C, 12 °C, 16 °C, 20 °C, or 37 °C.

	37 °C	20 °C	16 °C	12 °C	8 °C
**LB**	30.11	154.95	161.05	385.21	1167.30
**PCN**	79.98	176.81	225.56	410.09	1709.82
